# Development of a ghrelin receptor inverse agonist for positron emission tomography

**DOI:** 10.18632/oncotarget.27895

**Published:** 2021-03-02

**Authors:** Ralf Bergmann, Constance Chollet, Sylvia Els-Heindl, Martin Ullrich, Nicole Berndt, Jens Pietzsch, Domokos Máthé, Michael Bachmann, Annette G. Beck-Sickinger

**Affiliations:** ^1^Helmholtz-Zentrum Dresden-Rossendorf, Institute of Radiopharmaceutical Cancer Research, Dresden, Germany; ^2^Department of Biophysics and Radiation Biology, Semmelweis University, Budapest, Hungary; ^3^Institute of Biochemistry, Faculty of Life Sciences, Universität Leipzig, Leipzig, Germany; ^4^Tumor Immunology, University Cancer Center, Carl Gustav Carus Technische Universität Dresden, Dresden, Germany; ^5^National Center for Tumor Diseases, Carl Gustav Carus Technische Universität Dresden, Dresden, Germany; ^6^Faculty of Chemistry and Food Chemistry, School of Science, Technische Universität Dresden, Dresden, Germany; ^*^These authors contributed equally to this work

**Keywords:** cancer, prostate cancer, growth hormone secretagogue receptor (GHS-R), small animal imaging, copper-64

## Abstract

Imaging of Ghrelin receptors *in vivo* provides unique potential to gain deeper understanding on Ghrelin and its receptors in health and disease, in particular, in cancer. Ghrelin, an octanoylated 28-mer peptide hormone activates the constitutively active growth hormone secretagogue receptor type 1a (GHS-R1a) with nanomolar activity. We developed novel compounds, derived from the potent inverse agonist K-(D-1-Nal)-FwLL-NH_2_ but structurally varied by lysine conjugation with 1,4,7-triazacyclononane,1-glutaric acid-4,7-acetic acid (NODAGA), palmitic acid and/or diethylene glycol (PEG2) to allow radiolabeling and improve pharmacokinetics, respectively. All compounds were tested for receptor binding, potency and efficacy *in vitro*, for biodistribution and -kinetics in rats and in preclinical prostate cancer models on mice. Radiolabeling with Cu-64 and Ga-68 was successfully achieved. The Cu-64- or Ga-68-NODAGA-NH-K-K-(D-1-NaI)-F-w-L-L-NH_2_ radiotracer were specifically accumulated by the GHS-R1a in xenotransplanted human prostate tumor models (PC-3, DU-145) in mice. The tumors were clearly delineated by PET. The radiotracer uptake was also partially blocked by K-(D-1-Nal)-FwLL-NH_2_ in stomach and thyroid. The presence of the GHS-R1a was also confirmed by immunohistology. In the arterial rat blood plasma, only the original compounds were found. The Cu-64 or Ga-68-NODAGA-NH-K-K-(D-1-NaI)-F-w-L-L-NH_2_ radiolabeled inverse agonists turned out to be potent and safe. Due to their easy synthesis, high affinity, medium potency, metabolic stability, and the suitable pharmacokinetic profiles, they are excellent tools for imaging and quantitation of GHS-R1a expression in normal and cancer tissues by PET. These compounds can be used as novel biomarkers of the Ghrelin system in precision medicine.

## INTRODUCTION

The growth hormone secretagogue receptor type 1a (GHS-R1a) is the known biological relevant receptor of the endogenous ligand and pleiotropic hormone Ghrelin (acronym *growth hormone release inducing*), which mediates a broad range of complex biological functions [1], such as regulation of the body weight, body composition and energy expenditure [2, 3]. Besides GHS-R1a, a truncated form of the receptor exists which is termed GHS-R1b [4, 5]. In contrast to GHS-R1a, GHS-R1b does not bind Ghrelin and is completely inactive. Ghrelin and its receptor GHS-R1a are widely expressed in normal tissues but also in various tumors, including human pituitary adenomas, endocrine neoplasms of the lung, stomach, pancreas, breast, ovarian cancer and prostate carcinomas [6, 7].

Physiologically, Ghrelin is mainly involved in the positive regulation of energy homeostasis, hunger and body weight gain. The orexigenic mode of action of Ghrelin is well established and occurs via the activation of NPY/AgRP neurons in the hypothalamic arcuate nucleus [8, 9]. The GHS-R1a exhibits unusual high constitutive activity [10, 11] with ∼50% of its maximal capacity in the absence of the agonist (Ghrelin) GHS-R1a induces constant appetite and triggers food intake between meals [12]. In addition, Ghrelin receptors are involved in a series of biological processes including glucose homeostasis, GH-release, gastric motility, regulation of arterial pressure, bone metabolism, heart disease, and immune reactions. Moreover, Ghrelin is a potent anti-inflammatory mediator both *in vitro* and *in vivo* and a promising therapeutic agent in the treatment of cachexia, anorexia, age-related disorders [13], inflammatory diseases and injury. Through the MAPK signaling cascade Ghrelin can induce cell proliferation and could thereby play an important role in cancer. The growing knowledge about the interaction of Ghrelin with tumor cells suggests functional effects of Ghrelin on the tumor itself and the physiology of the body [14–17]. GHS-R1a is expressed in the prostate cancer cell lines PC-3 [18–20], DU-145 [21] and LnCAP [22, 23] and these cells can also secrete mature Ghrelin. These cells therefore represent a suitable model for *in vitro* experiments and *in vivo* studies as xenograft tumor models [24–26].

Interestingly, Ghrelin is the only known peptide modified with an O-linked octanoyl side group, which occurs on its third serine residue [27]. This modification is crucial for the physiological effects of Ghrelin including regulation of feeding, adiposity, and insulin secretion. The octanoylation is mediated by Ghrelin O-acyl transferase (GOAT). GOAT is a conserved orphan membrane-bound O-acyl transferase (MBOAT) that specifically octanoylates serine-3 of the Ghrelin peptide. Transcripts of both, GOAT and Ghrelin, occur predominantly in stomach and pancreas. GOAT is conserved across vertebrates, and genetic disruption of the GOAT gene in mice leads to complete absence of acylated Ghrelin in circulation. The occurrence of Ghrelin and GOAT in stomach and pancreas tissues demonstrates the relevance of GOAT in the acylation of Ghrelin and further implicates acylated Ghrelin in pancreatic function. GOAT should be taken into account as an additional binding site *in vivo* [28–30].

Although widely studied as a promising drug target, our knowledge about Ghrelin signaling, behavior, dynamic interactions with its receptor and functional receptor expression *in vivo* is still limited and basic bioscientific research is warranted to further evaluate the safety and benefits of Ghrelin drug treatment in patients with cancer [31–33]. *In vivo* imaging of the Ghrelin receptor should help to improve our understanding of its mode of action and might become a powerful tool for diagnosis and drug development [26, 34]. So far, only few groups tried to develop probes for PET and optical imaging of Ghrelin receptors [26, 35–40]. In particular the group of Lewis and colleagues started the research and development of PET radiotracers for imaging of the Ghrelin receptor [26, 37, 38, 41]. The emergence of accessible imaging techniques such as small animal PET could be very valuable to provide *in vivo* pharmacokinetic information all along the drug discovery process [38, 41–46].

In order to develop imaging probes targeting the Ghrelin receptor, the inverse agonist radiotracer ^68^Ga-NODAGA-KwFwLL-NH_2_ 1 was previously designed for PET imaging (Scheme 1). Despite a high metabolic stability and a broad biodistribution in rats, its poor potency prevented its use for further receptor dynamic studies or therapeutic application [46, 47]. More recently, the hexapeptide K-(D-1-Nal)-FwLL-NH_2_ 2 was developed and showed a very high inverse agonist potency toward the Ghrelin receptor as supported by the significantly decreased food intake in rats [47].

The focus of the present study was to develop potent inverse agonist radiotracers targeting the Ghrelin receptor as potential imaging and therapeutic agent. The *in vivo* accumulation of the radiotracer has been used as biomarker in diagnostics and therapy control. The observed agonistic ligand-mediated internalization of the GHSR-1a [48] could help to extend the retention of the small molecular ligand in the target tissue. Therefore, the heptapeptide KK-(D-1-Nal)-FwLL-NH_2_ 2 was functionalized on solid support with Palmitic acid and 1,4,7-triazacyclononane,1-glutaric acid-4,7-acetic acid (NODAGA) and radiolabeled with ^64^Cu, (half-life 12.701 hours, decays by 17.86% positron emission, and 0.653 MeV positron energy). This tracer was chosen, because it is beneficial for high-resolution small animal imaging with longer observation time. Furthermore, ^68^Ga was selected (half-life 67.629 minutes, 89% positron emission, 1.9 MeV positron energy), a generator radionuclide with optimal characteristics for clinical application, but shorter observation time and lower image resolution in the preclinical setting. The corresponding PEGylated and palmitoylated analogues were designed in order to study the influence of such modifications on radionuclide activity distribution and *in vivo* behavior of the radiotracers, and to improve pharmacokinetics [48].

Here, we report on the *in vitro* and preclinical *in vivo* suitability of selected NODAGA-Ghrelin receptor inverse agonists as radiotracers. Rats and mice were used as standard radiopharmacological models with respect to biodistribution, metabolic stability and PET imaging assays. Furthermore, we demonstrated specific binding to GHS-R1a and imaging of tumors and normal tissues expressing the Ghrelin receptor with ^64^Cu^2+^- and ^68^Ga^3+^-radiolabeled inverse agonist NODAGA-KK-(D-1-NaI)-FwLL-NH_2_ in xenografted prostate tumor models in mice.

## RESULTS

### Synthesis of Ghrelin inverse agonist derivatives and conjugation with NODAGA for labeling with metal-isotopes

**Figure 1:**
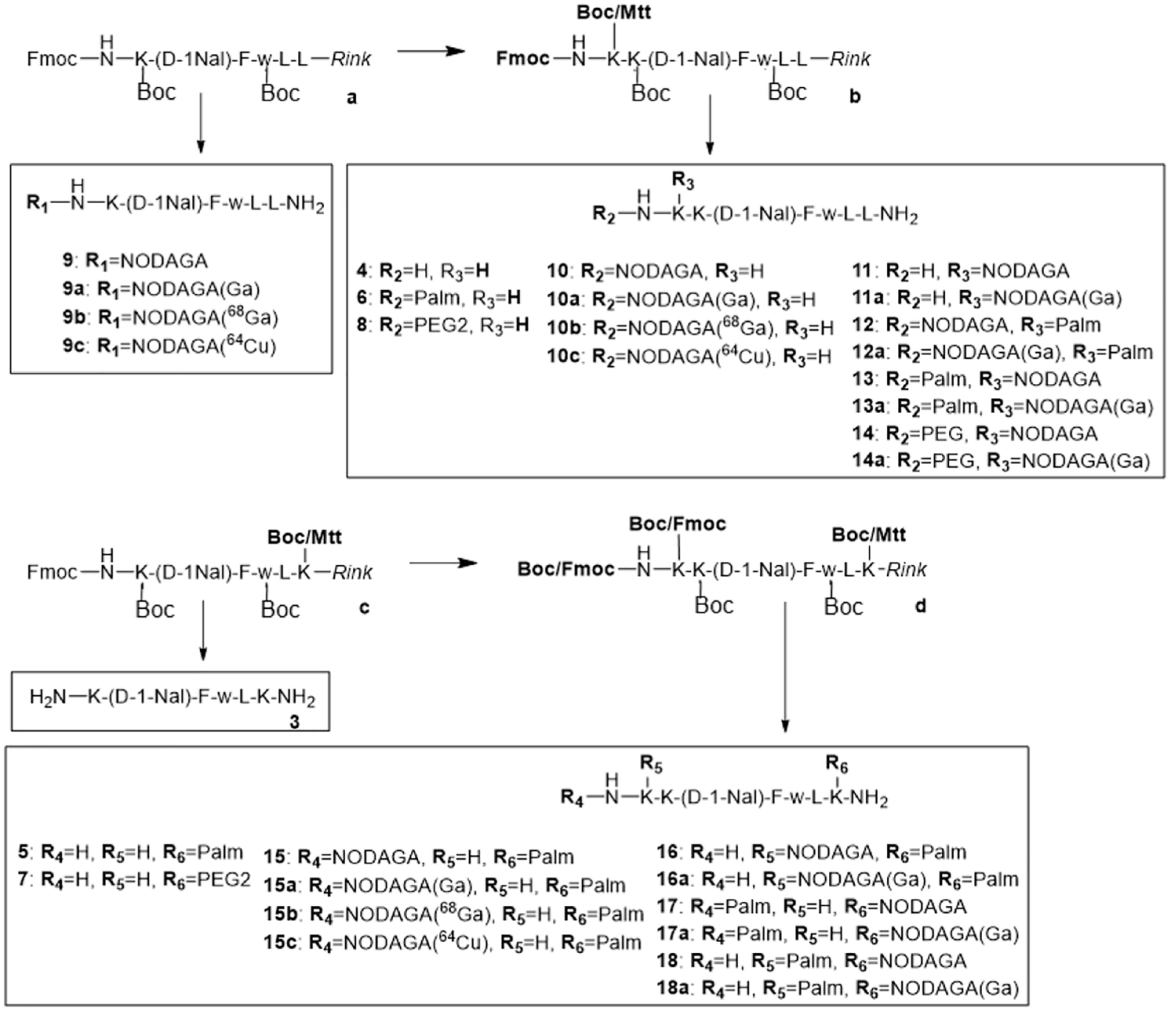
Synthesis of peptides 3–18. All peptides were synthesized on solid support using the Fmoc/tBu strategy and Mtt protecting groups for orthogonal side-chain modifications.

Novel Ghrelin inverse agonist derivatives were synthetized and conjugated with and without Palmitic acid. To apply these compounds for *in vivo* imaging it was necessary to attach a chelator to these molecules allowing us the binding of ^nat^Ga^3+^, ^68^Ga^3+^, and ^64^Cu^2+^ for *in vitro* testing and the *in vivo* imaging with PET. The optimal chelator for these metal isotopes was NODAGA that was attached at various positions in the molecules. As schematically summarized in Figure 1, eighteen Ghrelin inverse agonist derivatives were synthesized on a Rink amide resin (Table 1). The peptides 3–18 were purified (> 95%,) by RP-HPLC. Identity was confirmed by mass spectrometry. Prior to the *in vivo* experiments, a TFA-HCl exchange was performed for conjugates 9, 10 and 15 by incubating the peptides three times with diluted HCl and subsequent lyophilization. Integrity and purity of all compounds were controlled at each step by MS and HPLC.

**Table 1:** *In vitro* activity of peptides 2-18a in an inositol phosphate turnover assay

No.	Sequence	EC_50_ (nM)	pEC_50_ ± SEM	x-fold over No. 2	Δ_*eff*_ (%)
**2**	K-(D-1-Nal)-FwLL-NH_2_	4.5	8.4 ± 0.05	1	57 ± 2
**3**	K-(D-1-Nal)-FwLK-NH_2_	162.0	6.8 ± 0.07	36	42 ± 1
**4**	KK-(D-1-Nal)-FwLL-NH_2_	13.0	7.9 ± 0.09	3	63 ± 2
**5**	K-(D-1-Nal)-FwLK(Palm)-NH_2_	3.5	8.5 ± 0.07	1	85 ± 2
**6**	Palm-KK-(D-1-Nal)-FwLL-NH_2_	11.0	8.0 ± 0.06	2	86 ± 2
**7**	K-(D-1-Nal)-FwLK(PEG2)-NH_2_	166.0	6.8 ± 0.10	37	61 ± 3
**8**	PEG2-KK-(D-1-Nal)-FwLL-NH_2_	499.0	6.3 ± 0.08	111	85 ± 3
**9a**	[^nat^Ga]Ga-NODAGA-K-(D-1-Nal)-FwLL-NH_2_	64.8	7.2 ± 0.10	14	88 ± 8
**10a**	[^nat^Ga]Ga-NODAGA-KK-(D-1-Nal)-FwLL-NH_2_	34.7	7.5 ± 0.07	8	98 ± 4
**11a**	[^nat^Ga]Ga-NODAGA-K-(D-1-Nal)-FwLL-NH_2_	151.6	6.8 ± 0.07	34	78 ± 4
**12a**	[^nat^Ga]Ga-NODAGA-K(Palm)-K-(D-1-Nal)-FwLL-NH_2_	20.3	7.7 ± 0.06	5	92 ± 4
**13a**	Palm-K[^nat^Ga]Ga-NODAGA-K-(D-1-Nal)-FwLL-NH_2_	28.7	7.5 ± 0.06	6	92 ± 3
**14a**	PEG2-K[^nat^Ga]Ga-NODAGA-K-(D-1-Nal)-FwLL-NH_2_	> 1000	-	> 1000	n.d.
**15a**	[^nat^Ga]Ga-NODAGA-KK-(D-1-Nal)-FwLK(Palm)-NH_2_	16.5	7.8 ± 0.05	4	89 ± 3
**16a**	K[^nat^Ga]Ga-NODAGA-K-(D-1-Nal)-FwLK(Palm)-NH_2_	13.6	7.9 ± 0.07	3	90 ± 4
**17a**	Palm-KK-(D-1-Nal)-FwLK[^nat^Ga]Ga-NODAGA-NH_2_	592.1	6.2 ± 0.10	132	85 ± 6
**18a**	K(Palm)-K-(D-1-Nal)-FwLK[^nat^Ga]Ga-NODAGA-NH_2_	266.9	6.6 ± 0.10	59	75 ± 6

EC_50_ values (mean values and pEC_50_ *± SEM) were obtained from at least two independent experiments in duplicates and indicate the potency of the peptide. Efficacy values (Δ*_eff_) are the mean ± SEM of |Efficacy_max_ *-* Efficacy_min_|.

Ten novel NODAGA containing compounds were synthetized and prepared for testing of the efficacy and affinity at the GHS-R1a.

### *In vitro* activity of Ghrelin inverse agonists in inositol phosphate turnover assay

*In vitro* inositol phosphate turnover assay was performed to evaluate the potency and efficacy of peptides 3–7 and 9a-18a using COS-7 cells transfected with the Ghrelin receptor. The substitution of Leu^6^ with Lys in peptide 3 resulted in a significant loss of potency and efficacy (EC_50_ = 162 nM, Δ_eff_ = 42%). In contrast, addition of a Lys at the *N*-terminus in peptide 4 induced only a slight loss of potency (EC_50_ = 13.0 nM) but maintained efficacy of 63% compared to 2. Introduction of a palmitoyl group at the C-terminus led to the most potent and most efficient peptide K-(D-1-Nal)-FwLK(Palm)-NH_2_ (5), showing the same potency than the lead 2 (EC_50_ = 3.5 nM) and an efficacy of 85%. Palmitoylation at the *N*-terminal Lys linker led to peptide 6 which is only slightly less potent than 2 and equipotent to 4 (EC_50_ = 11.0 nM). The coupling of PEG2 at the C-terminal lysine (7) or at the *N*-terminal lysine linker (8) resulted in a drastic loss of potency (EC_50_ = 166 nM and 499 nM, respectively).

The biological activity of [^nat^Ga]Ga-NODAGA-chelates 9–18a as model compounds for the radiotracers was also evaluated. Direct introduction of [^nat^Ga]Ga-NODAGA at the *N*-terminus of the lead 2 resulted in a 14-fold drop in potency (9a, EC_50_ = 64.8 nM) and higher efficacy (Δ_eff_ = 88%). Addition of a Lys^1^ linker led to higher potency and efficacy when [^nat^Ga]Ga-NODAGA was branched at the *N*-terminus (10a, EC_50_ = 34.7 nM, Δ_eff_ = 98%) while both potency and efficacy were decreased when NODAGA was linked at Lys^1^ side chain (11a, EC_50_ = 152 nM, Δ_eff_ = 78%). Palmitoylated inverse agonist analogs 12–13a bearing both [^nat^Ga]Ga-NODAGA and palmitoylation at the Lys^1^ linker showed potencies in the nanomolar range (respectively EC_50_ = 20.3 nM and 28.7 nM) and high efficacies (Δ_eff_ = 92%). In contrast, the PEGylated inverse agonist derivative 14a presented a dramatic loss in potency with an EC_50_ reaching the micromolar range (EC_50_ = 1.8 ± 0.6 μM). Interestingly, compounds 15–16a with a *C*-terminal palmitoylation and an *N*-terminal [^nat^Ga]Ga-NODAGA presented the best potencies (EC_50_ = 16.5 and 13.6 nM, respectively) and high efficacies (Δ_eff_ = 89–90%), whereas the opposite configuration in derivatives 17–18a led to a 57- to 131-fold drop-in activity compared to the lead 2 (EC_50_ = 592.1 nM and 266.9 nM, respectively).

In the inositol phosphate turnover assay (Table 1), the efficacy of the NODAGA and Palm containing molecules decreased in the order 16a, 15a, 12a, 13a, 18a, 17a. The efficacy of NODAGA and non-Palm containing decreased in the order of 10a, 9a, 11a, 14a.

### *In vitro* affinity of Ghrelin inverse agonists at the Ghrelin receptor

Palmitic acid containing (12a, 15a) and non-containing (10a) compounds were selected for comparison of the affinity at the Ghrelin receptor. Competitive binding assays have been performed to evaluate the affinity of compounds 10a, 12a and 15a towards the Ghrelin receptor using ^125^I-His-Ghrelin with high molar activity and COS-7 cells stably transfected with the Ghrelin receptor (Table 2) [49]. Although lower than Ghrelin (K_i_ = 0.53 ± 0.03 nM) and the lead 2 (K_i_ = 4.9 ± 0.8 nM), all derivatives displayed affinities in the nanomolar range with K_i_ of 30.3 ± 7.5 nM, 66.9 ± 25.7 nM, and 11.0 ± 4.3 nM, respectively. Interestingly, the palmitoylated peptide 15a showed the best affinity at the receptor, with only a 2-fold reduction compared with the control 2. Hence, palmitoylation is beneficial when placed at the *C*-terminus (15a versus 10a) whereas it lowers the affinity of the tracer when introduced at the *N*-terminus (12a versus 10a).

**Table 2:** In vitro binding affinity of peptides

No.	Peptide	K_i_ ± SD [nM]	x-fold over Ghrelin	x-fold over 2	*n*
**Ghrelin**	GS-S(Oct)-FLSPEHQRVQQRKESKKPPAKLQPR-OH	0.53 ± 0.03	1	0.1	3
**10a**	[^nat^Ga]Ga-NODAGA-KK-(D-1-Nal)-FwLL-NH_2_	30.3 ± 7.50	57	6	2
**12a**	[^nat^Ga]Ga-NODAGA-K(Palm)-K-(D-1-Nal)-FwLL-NH_2_	66.9 ± 25.7	126	14	2
**15a**	[^nat^Ga]Ga-NODAGA-KK-(D-1-Nal)-FwLK(Palm)-NH_2_	11.0 ± 4.30	21	2	2

*In vitro* binding affinity of peptides 10a, 12a and 15a toward the Ghrelin receptor in competitive binding assay with ^125^I-His-Ghrelin. K_i_ values (mean ± SD) were obtained from concentration-response curves in two independent experiments in duplicates.

The affinity of the ^nat^Ga-peptides to the GHS-R1a is approximately two orders of magnitude lower that Ghrelin’s.

### Complexation with ^nat^Ga^3+^ and radiolabeling with [^64^Cu]Cu^2+^ and [^68^Ga]Ga^3+^

For *in vitro* and *in vivo* studies, the radiochemical yield (RCY) was > 95% after incubation at 90°C for 30 min with a molar activity equal or larger than 20 GBq × μmol^-1^. High amounts of radiolabeled conjugates were obtained (280 to 430 MBq) which allowed performing of biodistribution and PET experiments in parallel. Nevertheless, the high radiochemical purity (> 98%) and molar activity allowed the direct application of the 9b-c, 10b-c and 15b-c without further purification of the products. The experiments are summarized in Table 3. In the experiments the molar activities of the radiotracers were comparable for both radionuclides. The molar activity for 9c resulted from a lower specific activity of the ^64^Cu^2+^ provided. The highest molar activities were obtained with peptide 10, with average molar activities at time of injection of 38.6 ± 37.7 GBq/μmol and 43.4 ± 41.1 GBq/μmol (mean ± SEM) for ^68^Ga and ^64^Cu respectively.

**Table 3:** Molar activities

[^68^Ga]Ga^3+^- radiotracers	Molar activity (GBq/μmol)	*n*	[^64^Cu]Cu^2+^- radiotracers	Molar activity (GBq/μmol)	*n*
9b	19.7 ± 16.7	7	9c	6.3 ± 3.8	10
10b	38.6 ± 37.7	6	10c	43.4 ± 41.1	16
15b	16.1 ± 6.1	12	15c	16.6 ± 8.3	10

Molar activities of 9b, 9c, 10b, 10c, 15b and 15c at time of injection (mean ± SEM of *n* experiments).

All the compounds could be radiolabeled with molar activities suitable for radiotracer experiments *in vivo*.

### *In vivo* metabolic stability of radiolabeled Ghrelin inverse agonists in rats

**Figure 2:**
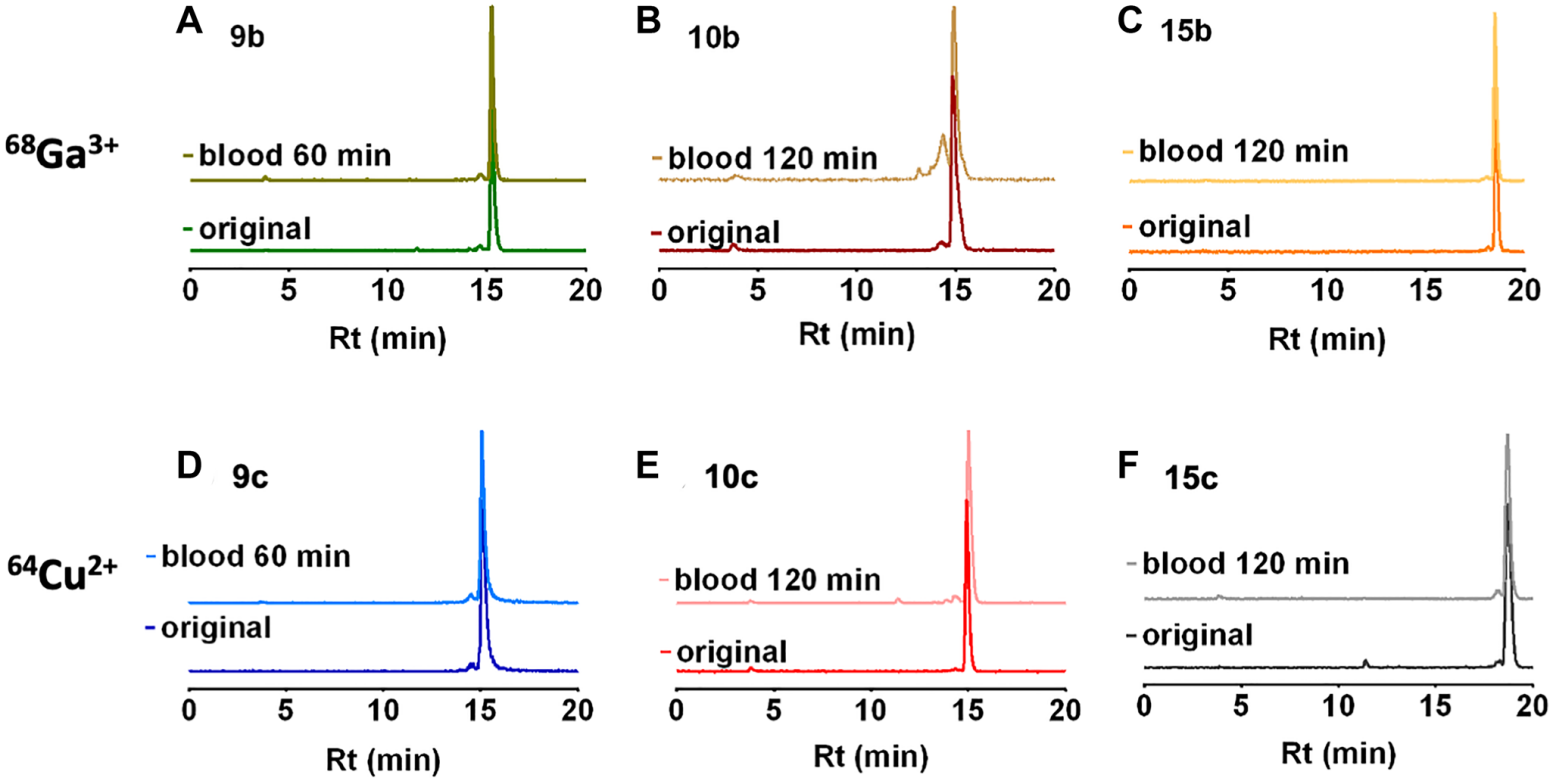
Radio-HPLC of rat blood plasma. Radio-HPLC of rat blood plasma at 0 and 60 or 120 minutes after single intravenous injection of the ^68^Ga-radiotracers (**A**–**C**) 9b, 10b and 15b and the ^64^Cu-radiotracers (**D**–**F**) 9c, 10c and 15c.

The metabolism of the radiolabeled peptides was studied in deproteinized arterial blood plasma of rats. Radio-HPLC chromatograms of [^68^Ga]Ga^3+^-radiotracers 9b, 10b, 15b and [^64^Cu]Cu^2+^-radiotracers 9c, 10c, and 15c are presented in Figure 2.

In the circulating blood, all radiotracers were mainly found as original compound and only traces of radioactive metabolites were detected with 10b. The chromatogram of 10b showed a minor metabolite at 120 min after injection. The distribution of intact 10c was further evaluated. At 1 hour after injection 14% of the original compound were found in the liver, 1% in the kidneys, and 6% in the urine. The structure of the metabolites was not further characterized.

As nearly no radioactive degradation products were observed in the arterial blood (Figure 2) we could therefore assume that the blood activity concentration reflect the original radiotracer with no or minor metabolite amounts. Consequently, concentration of the radiotracer peptide concentrations in the blood could be estimated according to their molar activity, injected activity, and the body weight (Table 4).

**Table 4:** Estimated radiotracer peptide concentrations in blood

Biodistribution					
[^68^Ga]Ga^3+^-radiotracers	C_*blood*_ (nM)	*n*	[^64^Cu]Cu^2+^-radiotracers	C_*blood*_ (nM)	*n*
9b	28 ± 24	14	9c	4.5 ± 0.9	16
10b	30 ± 16	14	10c	2.5 ± 1.3	15
15b	90 ± 6	15	15c	4.1 ± 2.4	15
**PET**					
**[^68^Ga]Ga^3+^-radiotracers**	**C_*blood*_ (nM)**	***n***	**[^64^Cu]Cu^2+^-radiotracers**	**C_*blood*_ (nM)**	***n***
9b	1100 ± 800	2	9c	570 ± 240	2
10b	530 ± 210	2	10c	270 ± 110	2
15b	670	1	15c	560 ± 310	2

Estimated peptide concentrations of the radiotracers in blood (directly after injection in the PET) and 5 min after injection in the biodistribution studies. The blood volume was calculated as 6.5% of the body weight.

It is important for comparison of the results of the biodistribution and the PET studies to know the injected peptide amounts. Under our experimental conditions and dependent on the physical half-life of the radioisotopes were the starting peptide concentrations in the blood lower for the ^64^Cu-labeled radiotracers compared to the ^68^Ga-labeled radiotracers. Comparing biodistribution and PET experiments the other factor that forced the injection of larger activity amounts in PET was the lower sensitivity of the imaging system in relation to the well counter used in biodistribution measurements. The consequence of approximately two orders of magnitude higher peptide concentration in the PET experiments was that the GHS-R1a were partially blocked by the radiotracer peptide itself and the blocking effects of the Ghrelin and the small peptide inverse agonist KK-(D-1-Nal)-FwLL-NH_2_ (named KKD) were smaller than in the biodistribution experiments. In the biodistribution experiments of 9c, 10c and 15c were the calculated radiotracer peptide to K_i_ ratios comparable to the ratio of Ghrelin level (0.09 - 0.22 nM) [50] to K_i_ (Ki = 0.53 ± 0.03 nM, Table 2) *in vivo* in lined rats.

The high metabolic stability and the low necessary tracer quantities should allow the quantification of the ghrelin receptors in the biodistribution studies. In the PET examinations, the amount of substance required is up to 100 times greater and thus will results in partial saturation of the receptors with the result of lower inhibiting effects in the competition experiments.

### Biodistribution of radiolabeled Ghrelin inverse agonists in rats

**Figure 3:**
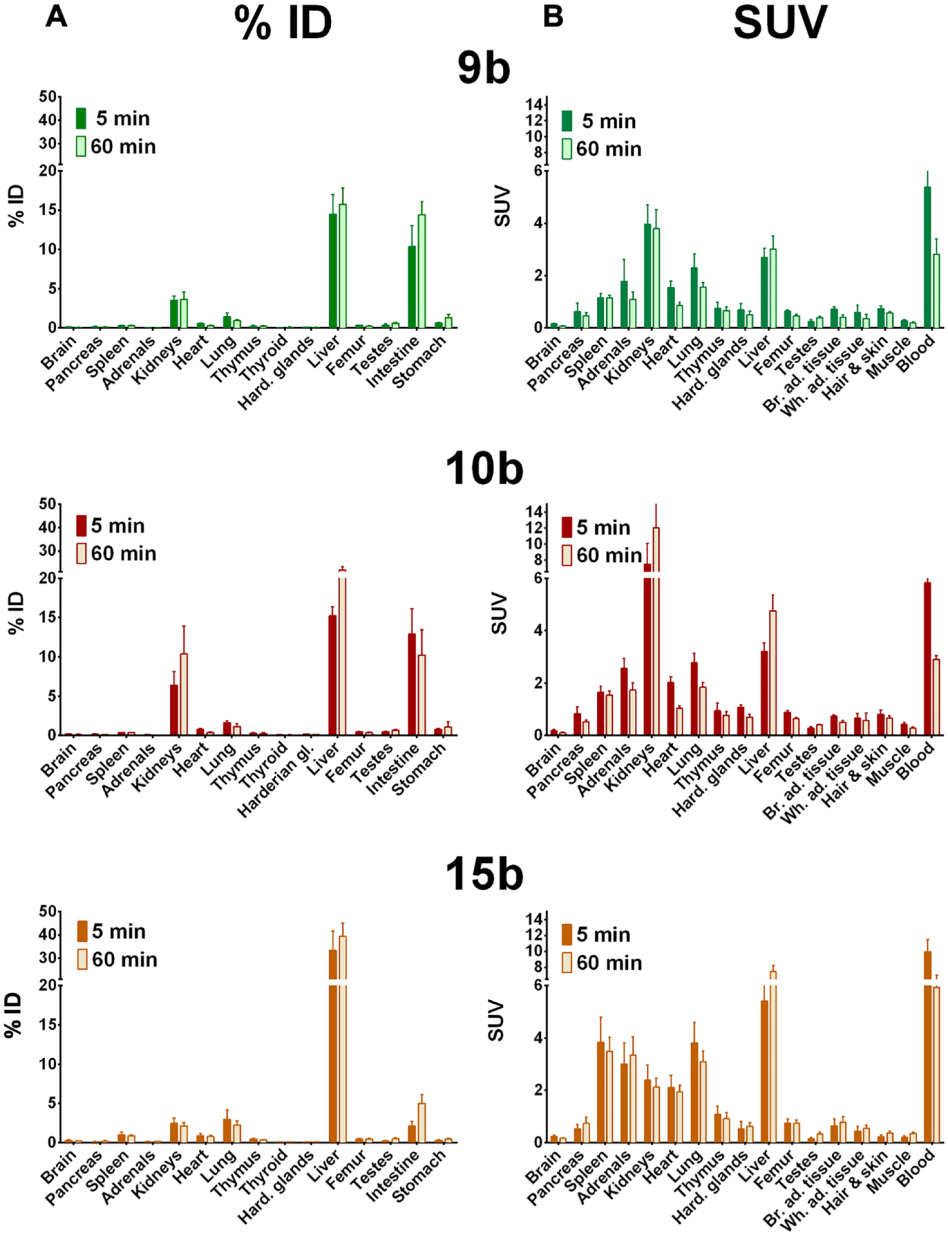
Biodistribution of ^68^Ga-radiotracers in healthy rats. Biodistribution of ^68^Ga-radiotracers 9b, 10b and 15b in healthy male Wistar rats after single intravenous injection at 5 and 60 min, the activity amounts in selected organs are expressed as percent of the injected dose (**A**, % ID) and the activity concentrations in the tissues are given as standard uptake values (**B**, SUV).

**Figure 4:**
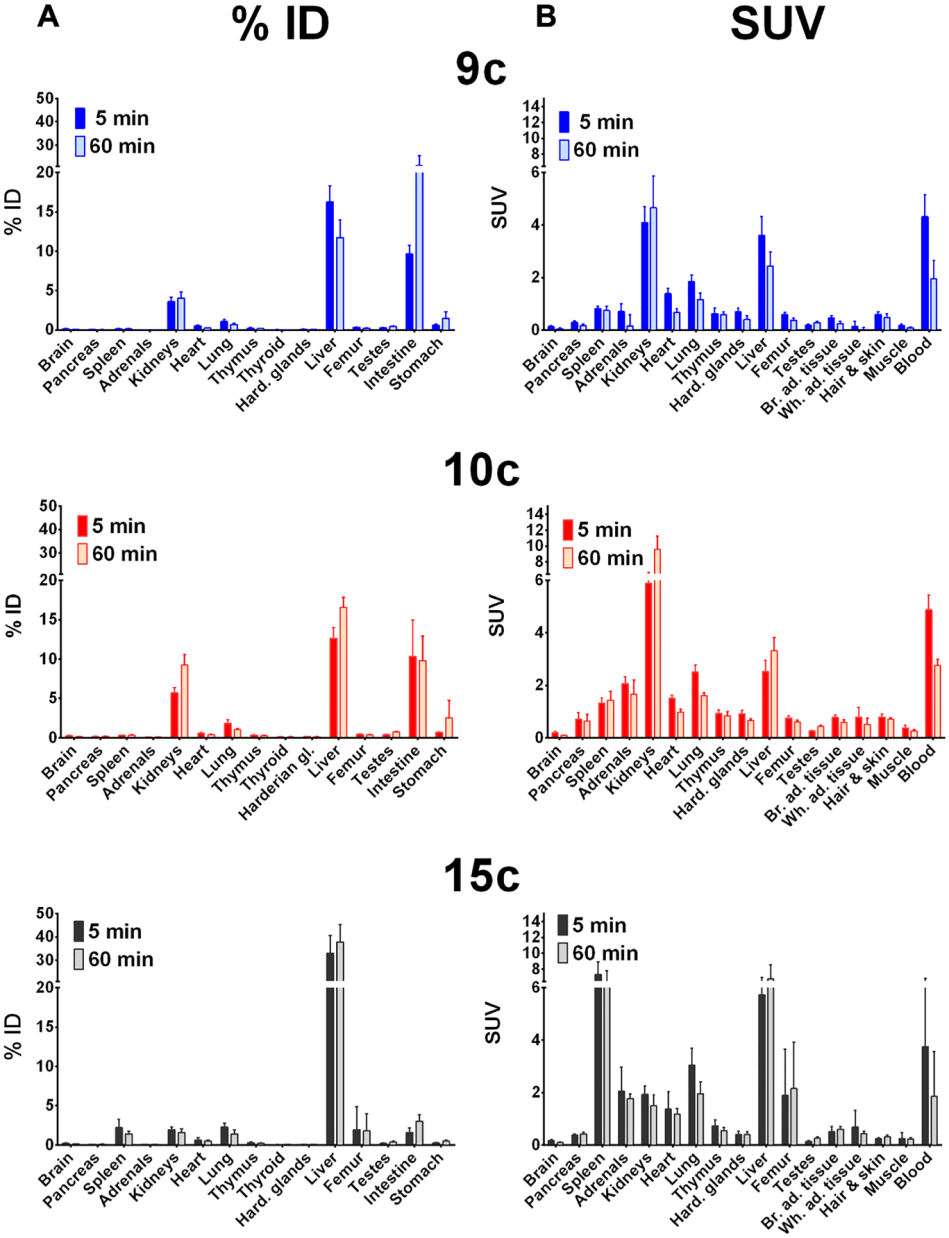
Biodistribution of ^64^Cu-radiotracers in healthy rats. Biodistribution of ^64^Cu-radiotracers 9c, 10c and 15c in healthy male Wistar rats after single intravenous injection at 5 and 60 min, the activity amounts in selected organs are expressed as percent of the injected dose (**A**, % ID) and the activity concentrations in the tissues are given as standard uptake values (**B**, SUV).

Biodistribution in distinct organs and tissues of healthy rats was measured at 5 and 60 min after single intravenous injection of the ^68^Ga- or ^64^Cu-radiotracers 9b-c, 10b-c, and 15b-c in rats (see ^68^Ga-tracers in Figure 3 and ^64^Cu-tracers in Figure 4).

In general, all compounds followed the tissue perfusion and were rapidly released from the tissues with minor or slow decrease of the radiotracer concentrations in most organs between 5 and 60 min. The accumulation and diffusion of the radiotracers in the tissues primarily depended on their concentration in arterial blood. Hence, 9b-c, 10b-c and 15c showed relative high levels in the blood after 5 min (SUV = 4–6, Figure 3 and 4B) that remained also after 60 min (SUV = 2–3). In contrast, the level of the ^68^Ga-labeled palmitoylated tracer 15b was remarkably elevated in blood with a SUV twice as high as the other radiotracers (from 10 to 6 within one hour).

Interestingly, biodistribution of ^68^Ga- and ^64^Cu-radiotracers followed nearly the same pattern. At 5 min after injection, ^68^Ga- and ^64^Cu-radiotracers showed only minor differences with the notable exception of ^68^Ga-15b, which was present in blood in higher concentration than ^64^Cu-15c as described. At 60 min higher amounts of ^68^Ga-radiotracers 9b and 15b were found in most organs compared to ^64^Cu-radiotracers 9c and 15c except in intestine where surprisingly high amount of 9c was observed and in the spleen that showed higher amount of 15c. In contrast, no significant difference in the amounts of ^68^Ga-10b and ^64^Cu-10c was detected.

In addition, all radiotracers presented a major hepatobiliary and intestinal elimination (Figure 3A and 3B). Concentration of 9b stayed constant in liver (15% ID) while increased concentrations of 10b was observed within one hour (15 to 22% ID) reflecting liver accumulation. The palmitoylated tracer 15b exhibited the highest but constant concentration in the liver within one hour after injection (± 35% ID) probably because of the high lipophilicity of the palmitoyl chain. In intestine, uptake of 9b increased from 10 to 14% ID whereas concentration of 10b stayed constant (10–13% ID) and concentration of 15b was remarkably low (< 5% ID). In contrast to hepatobiliary and intestinal clearance, a minor renal clearance was observed for all tracers. Hence, kidney uptake was low for tracers 9b and 15b (< 4% ID) and moderate for 10b although concentrations of the latter one increased from 6 to 10% ID within one hour.

Among other organs, the concentration of the palmitoylated analogs in the spleen was remarkable. 15b showed high but constant concentration (3.5 to 3.8 SUV) while 15c reached the highest uptake of 6.5 to 7.4 SUV within one hour. At last, although low, the brain uptake was not negligible. An approximately 2-fold decrease in uptake of all radiotracers was observed within one hour after injection. The radiotracers were still detectable in brain with 0.06 to 0.1 SUV for 9b-c, 10b-c, and 15c, and 15b reaching the highest concentration with 0.16 SUV.

**Figure 5:**
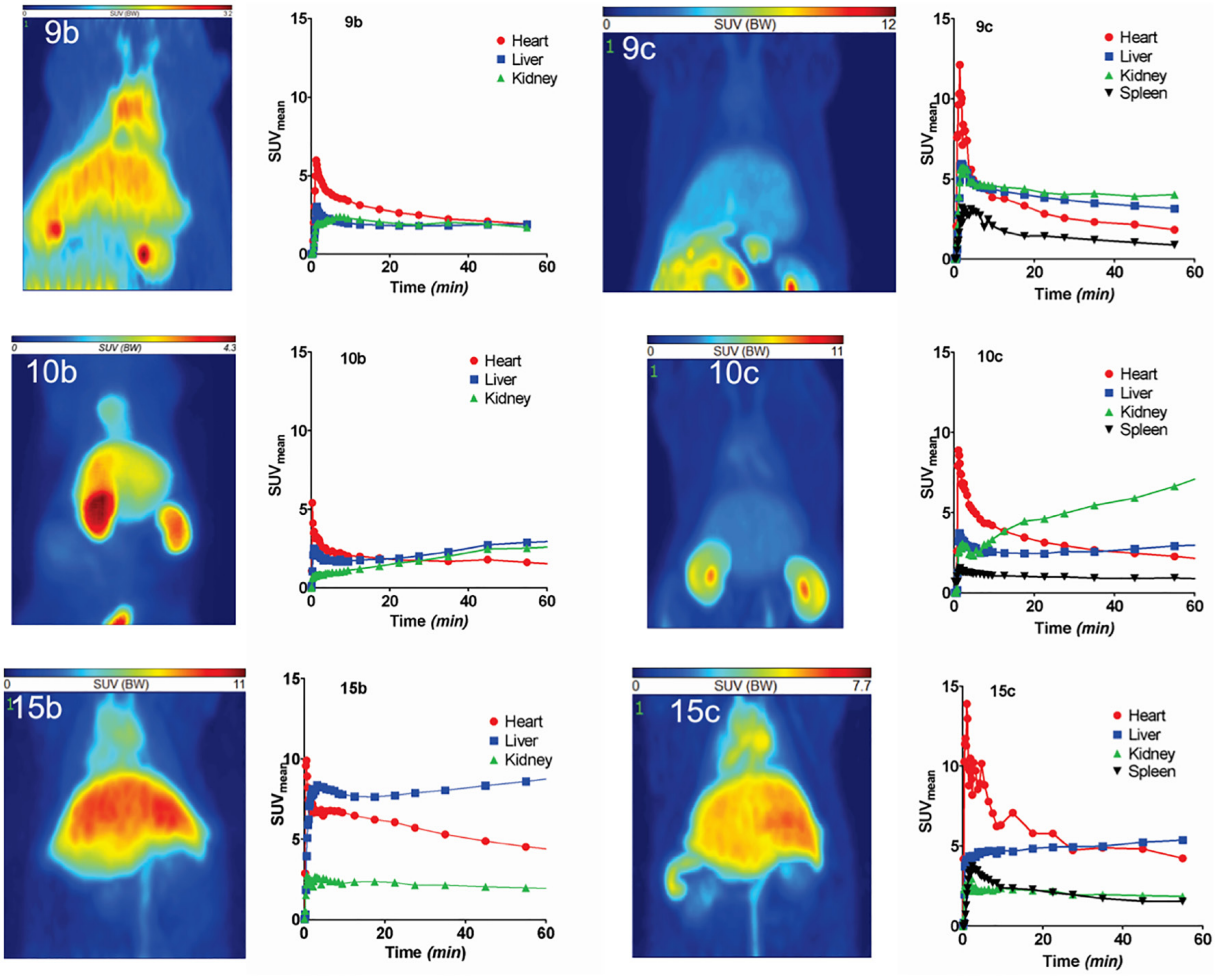
PET studies of ^68^Ga- and ^64^Cu-labeled radiotracers in healthy rats. PET studies of radiotracers 9b, 9c, 10b, 10c, 15b and 15c in Wistar rats. The images represent the maximum intensity projections of the radiotracer distribution 1 h after injection. The figures show the time-activity curves in the clearly visible and definable organs (dynamic measurement over one hour). Values are given as SUVmean.

**Figure 6:**
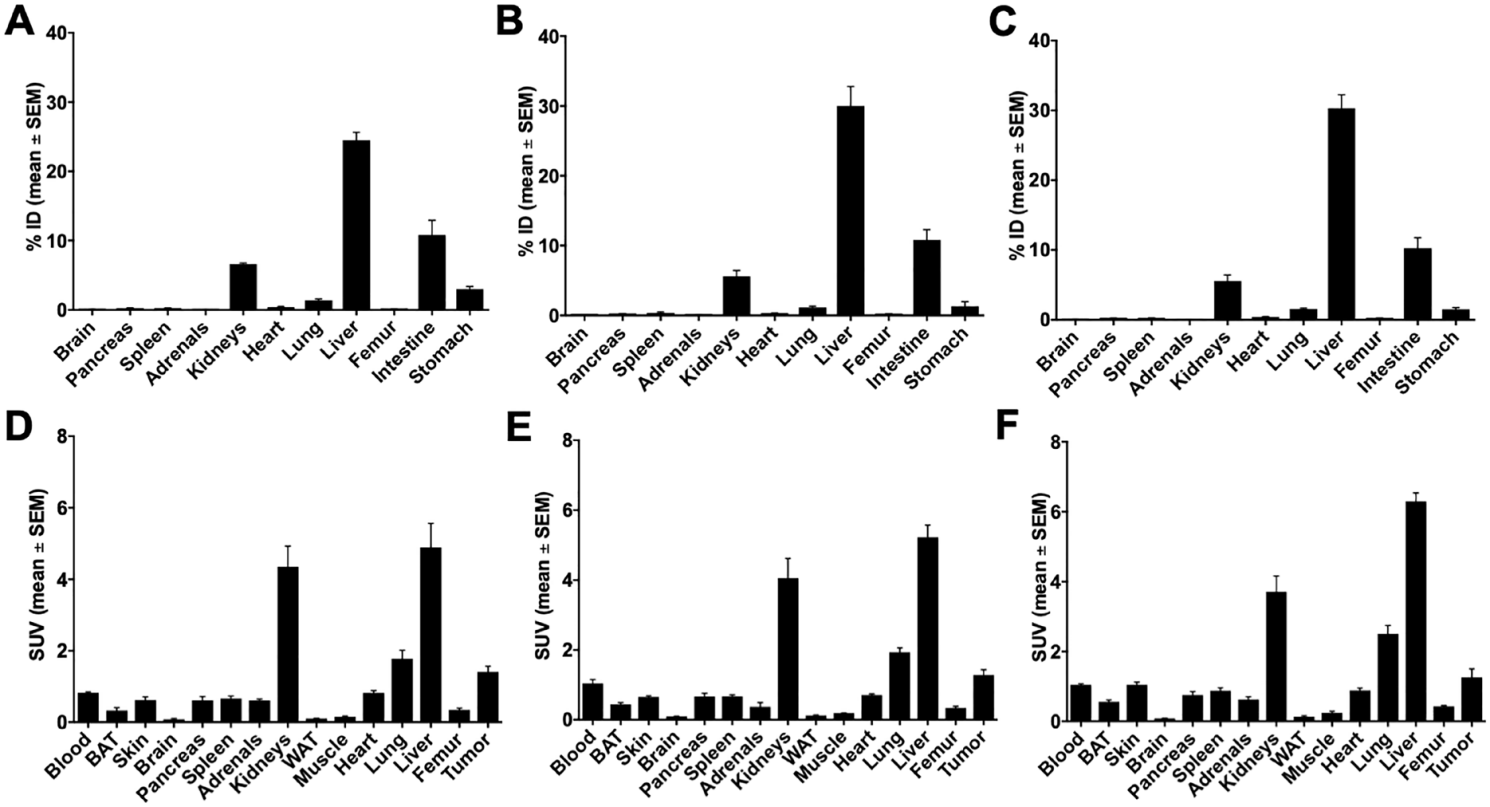
Biodistribution of 10c in DU-145 tumor bearing NMRI nu/nu mice 60 min p.i. Biodistribution of 10c in DU-145 tumor bearing NMRI nu/nu mice 60 min p.i. (**A**, **D**) Control. (**B**, **E**) Blocked with 1 mg/kg body weight Ghrelin. (**C**, **F**) Blocked with 1 mg/kg body weight KKD. Values are presented as % ID and SUV as mean ± SEM of three animals.

**Figure 7:**
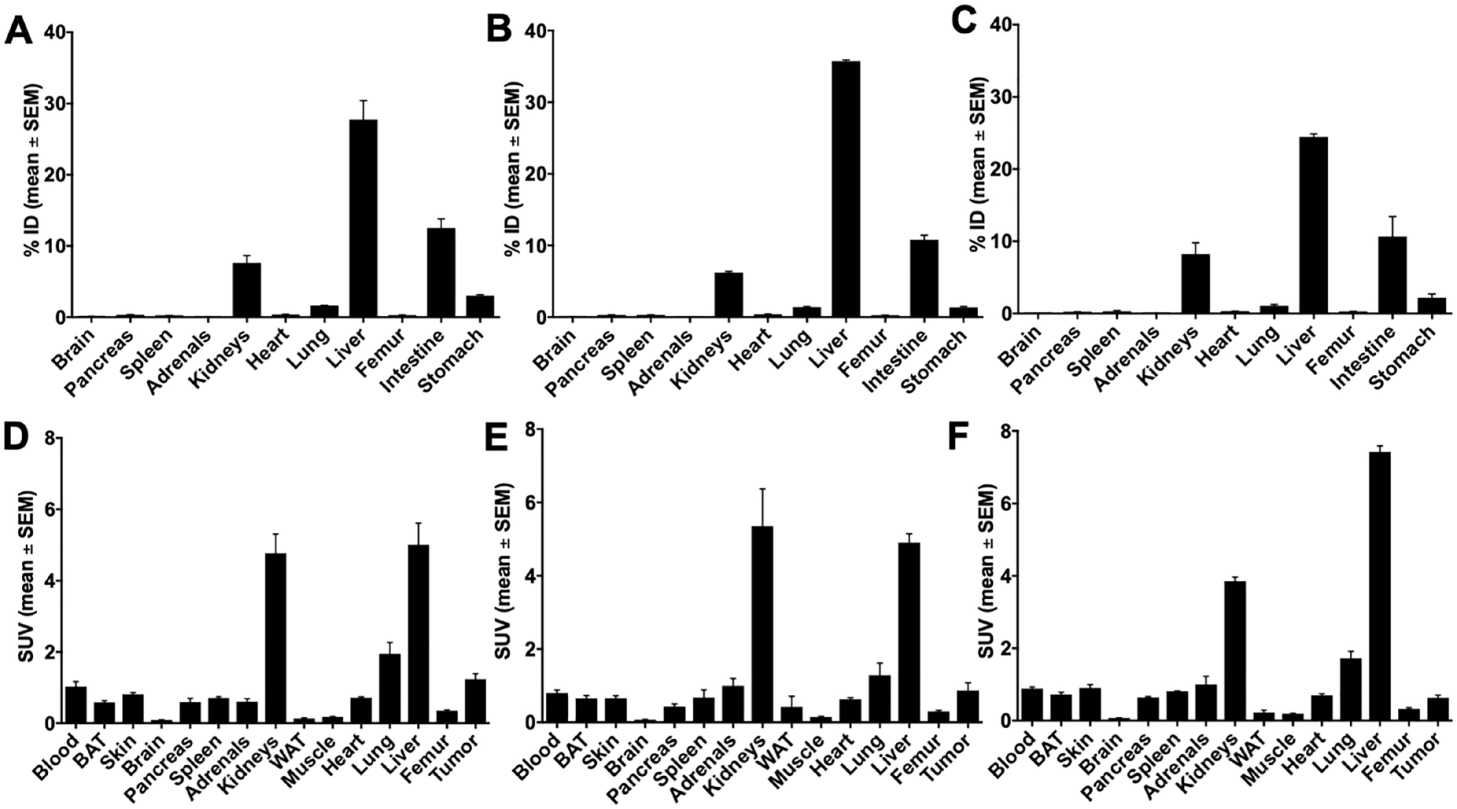
Biodistribution of 10c in PC-3 tumor bearing NMRI nu/nu mice 60 min p.i. Biodistribution of 10c in PC-3 tumor bearing NMRI nu/nu mice 60 min p.i. (**A**, **D**) Control. (**B**, **E**) Blocked with 1 mg/kg body weight Ghrelin. (**C**, **F**) Blocked with 1 mg/kg body weight KKD. Values are presented as % ID and SUV as mean ± SEM of three animals.

The three radiotracers were differently accumulated in the stomach, a natural GHS-R1a expressing organ. The activity amounts were comparable for rat and mouse (Figures 3–7). The 10c amount in the stomach was larger than that of the other compounds. This accumulation of 10c in an organ expressing the Ghrelin receptor could be an indication of specific binding to the GHS-R1a.

The direct comparison of the biodistribution of 10b and 10c in healthy Wistar rats shows that the liver uptake of 10b and 10c is relatively fast and increased only slowly, and the ^68^Ga compound had a slightly higher liver uptake than the ^64^Cu labeled compound. This demonstrates that the liver uptake is primarily caused by the ghrelin derivative itself and not by the copper release from the chelate complex with NODAGA.

### PET imaging and kinetics of radiolabeled Ghrelin inverse agonists in rats

Representative maximum intensity projections of the activity distribution at 60 min after injection and time-activity curves in kidneys, liver, heart and spleen of all tracers are shown in Figure 5. The results were consistent with biodistribution studies. 9b and 9c were broadly distributed in all organs and sustained concentrations were observed in heart, kidney and liver. High concentrations of 10b are visible in liver, kidney and circulation, whereas 10c was mainly retained in kidney after one hour. Images of the palmitoylated tracers 15b and 15c highlighted their major hepatic clearance but also showed elevated activity in the circulation (visible in heart and jugular veins) and low concentration in the kidney. In addition, accumulation of the ^64^Cu-labeled radiotracers (9c, 12c, 15c) in the spleen was detectable.

The PET experiments show relative slow elimination from the blood and accumulation in liver, kidneys and spleen. The ^64^Cu-images show more details in PET in comparison to the ^68^Ga-images.

### Biodistribution of [^64^Cu]Cu-NODAGA-NH-K-K-(D-1-NaI)-F-w-L-L-NH_2_ (10c) in DU-145 and PC-3 tumor bearing mice

The ^64^Cu-labeled 10c was selected for the further biopharmaceutical evaluations because of the high molar activity of the ^64^Cu-compounds, the best imaging properties of ^64^Cu, the lower efficacy, awaiting lower pharmacodynamic side effects, and the simpler synthesis without Palm conjugation, and the resulting lower lipophilicity. The lipophilicity is indicated from the earlier elution in the C-18 chromatography. The Figure 6 show the DU-145 and the Figure 7 the PC-3 biodistribution data.

In general, the activity concentration (SUV, mean ± SEM) was highest in the liver (3.90 ± 0.87), kidneys, tumor, heart, lung, testes, ovaries, spleen, blood, uterus, skin, pancreas, adrenals, femur, brown adipose tissue (BAT), brain, white adipose tissue (WAT), and lowest in muscle (0.29 ± 0.07). Between all these tissues, only the skeleton muscle was not reported in the literature to contain significant amounts of GHS-R. The skeleton muscle was therefore used as reference tissue for the background.

**Figure 8:**
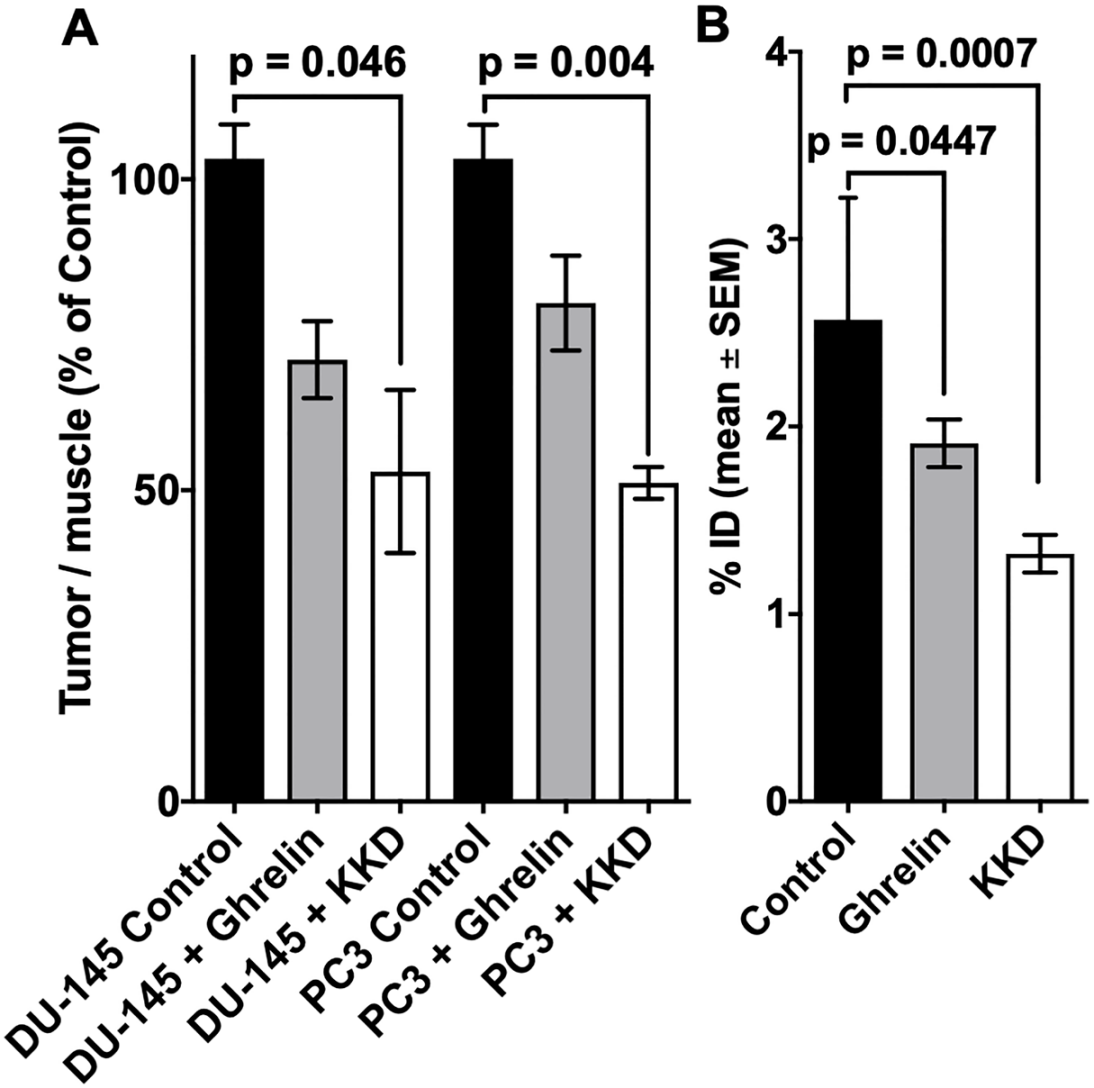
Blocking of 10c accumulation in tumors and stomach. The accumulation of 10c in the tumors (**A**) and stomach (**B**) at 60 min p.i. of Control and blocked with 1 mg/kg body weight Ghrelin or KKD in mice xenografted with DU-145 or PC-3 tumors. The tumor to muscle ratio is presented as % of Control and the stomach activity as % ID. All values are expressed as mean ± SEM of three animals in each tumor (A) and six animals’ stomachs (B).

The comparison of the activity amounts and concentrations between control and competition experiments showed blocking effects in the tumors and stomach (Figure 8). In both tissues was the accumulation of 10c decreased by Ghrelin and KKD. The largest blocking effect was observed at one hour after injection in the PC-3 tumors with KKD. The tumor to muscle ratios, calculated as percent of control, were decreased with Ghrelin and KKD for PC-3 to 83.4 ± 18.4% and 51.2 ± 4.5% and the values for the DU-145 to 71.0 ± 10.7% and 59.7 ± 30.2%, respectively. In the PET experiments at one hour were the tumor to muscle ratios in PC-3 tumors decreased after blocking with KKD to 31.7 ± 0.7% and to 43.7 ± 0.4% of Control. On the one hand, a significant decrease of 10c accumulation was also detected in testes, brain, and heart. On the other hand, a significant increase was observed for adrenals, lung, BAT and liver. This “blocking” pattern can be explained as a result of a “sink effect” of the broad distribution of GHSRs and other Ghrelin binding sites in the organism. In blocking experiments, the high concentration of Ghrelin and KKD after the simultaneous injection in the blood plasma liberates 10c from a number of binding sites in the organism. This increases the amount of free biologically available 10c and as result the activity was also increased in the adrenals, lung and liver, in organs with relatively large fractional blood volume. Nevertheless, there could be also compensatory effects like in spleen with no changes and in the BAT with low blood volume but with an increase of 10c concentration. This complex blocking effect pattern is a result of the wide distribution of the GHS-R1a in the organism. This demonstrates that 10c should allow tracing the Ghrelin receptor distribution in many tissues.

### PET imaging and kinetics of [^64^Cu]Cu-NODAGA-NH-K-K-(D-1-NaI)-F-w-L-L-NH_2_ (10c) in DU-145 and PC-3 tumor bearing mice

**Figure 9:**
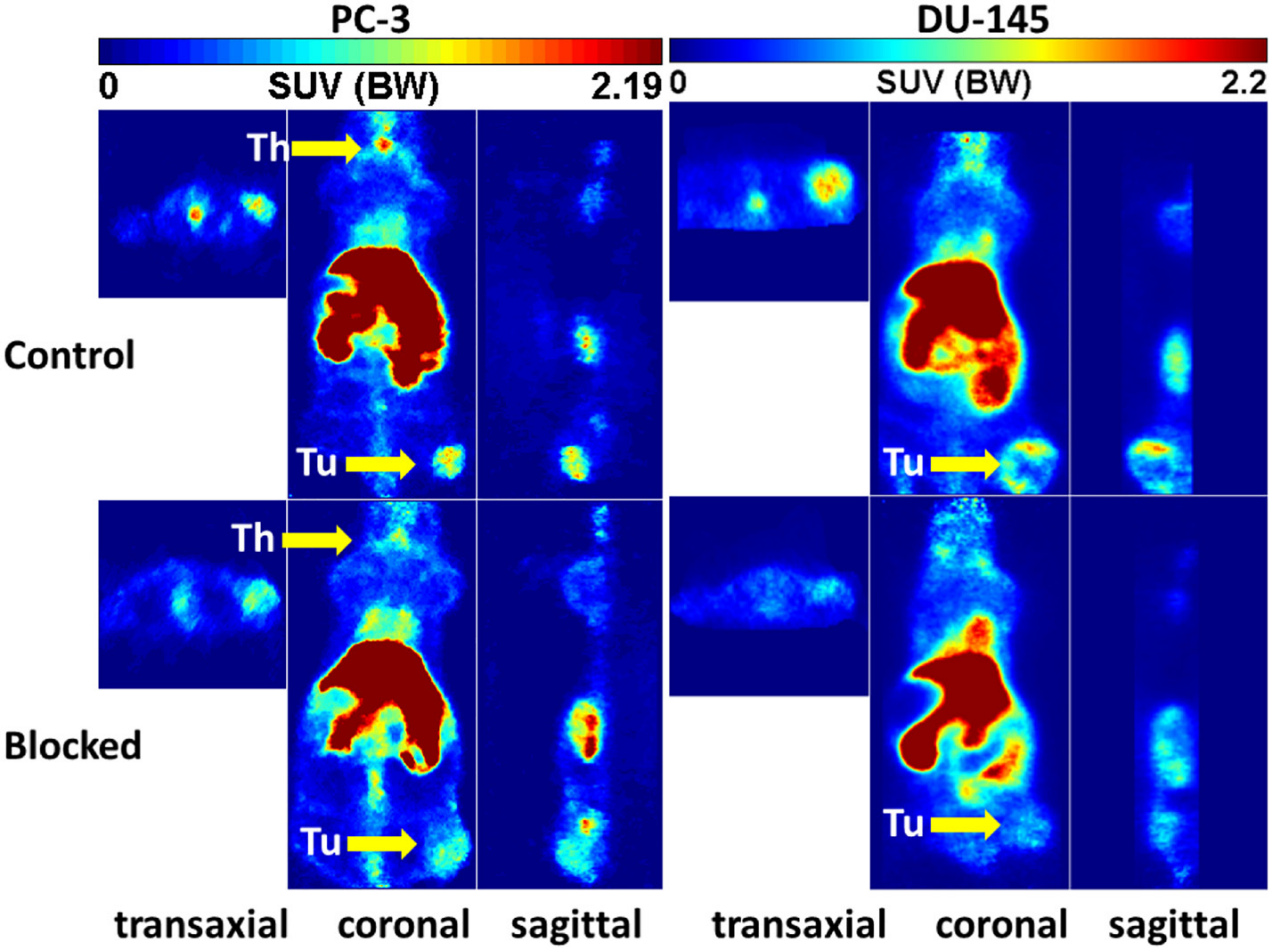
Representative orthogonal sections of PET studies. Representative orthogonal sections of PET studies with 10c in of PC-3 (left) and DU-145 (right) tumor-bearing NMRI nu/nu mice at 90 min midframe time (images were summarized from 60 min to 120 min). Typical Control (upper) and blocked mice (lower) by simultaneous injection of 1 mg/kg body weight of KKD are shown Abbreviations: Th, thyroid; Tu, tumor.

**Figure 10:**
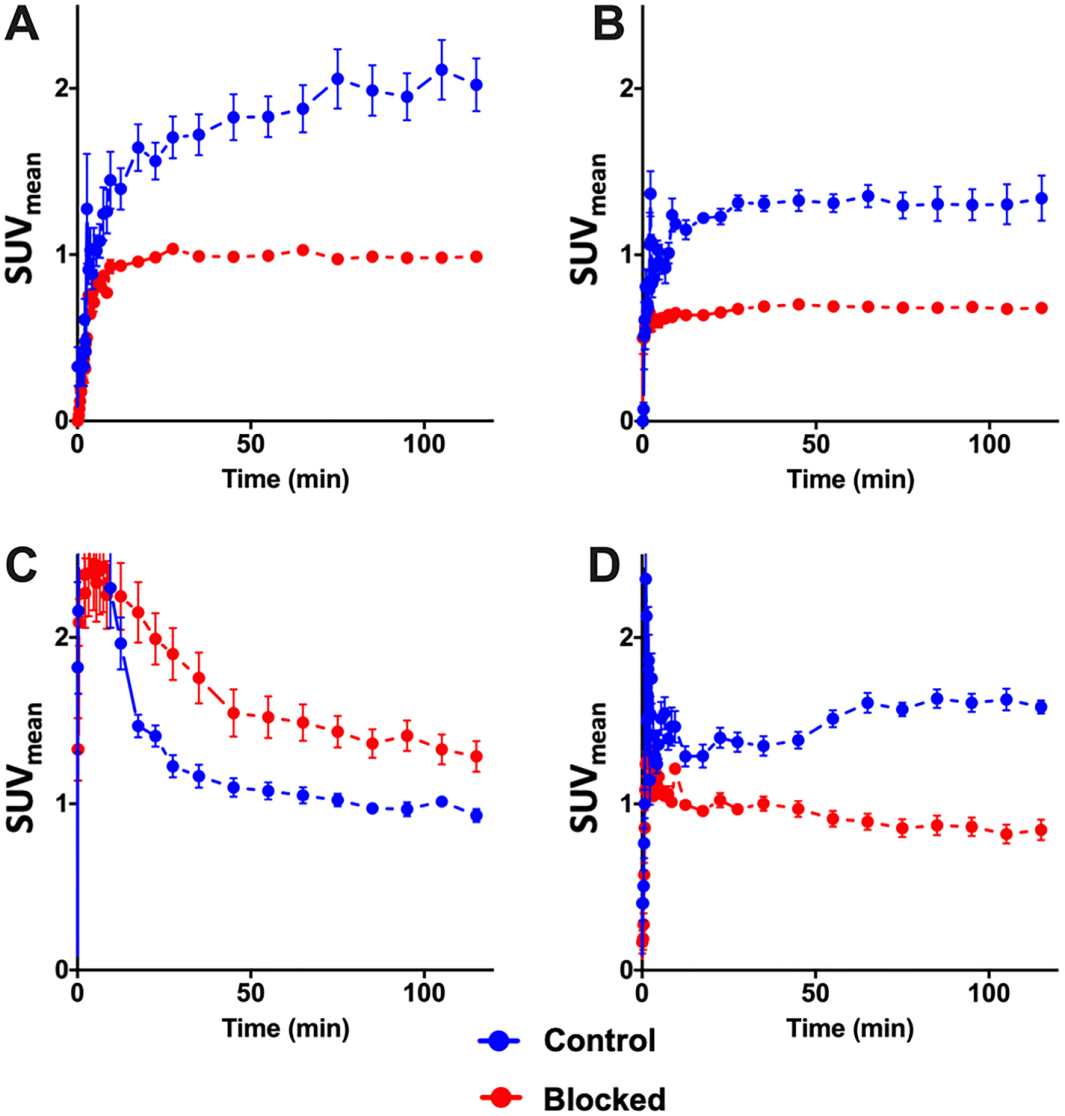
PET time activity curves. PET time activity curves of 10c in PC-3 (*n* = 3) (**A**) and DU-145 (*n* = 3) (**B**) tumors, in blood (**C**) (*n* = 6) and in the thyroids (**D**) (*n* = 6) of these animals. 10c was intravenously injected in the animals without (Control, blue) or with simultaneous injection (Blocked, red) of 1 mg/kg body weight of KKD. The curves show the mean ± SEM (SUV).

**Figure 11:**
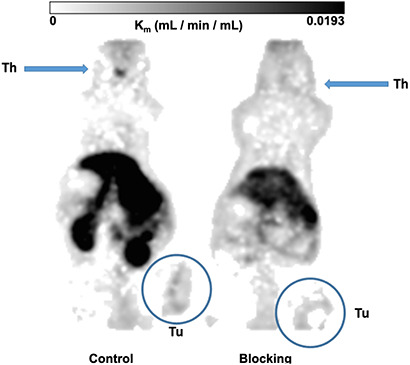
Metabolic trapping rate (Patlak K_m_) images. The metabolic trapping rate images (Patlak K_m_) of PC-3 tumor bearing mice after single intravenously injection of 10c were calculated from the dynamic PET studies and the image derived blood activity curves. The Control (left) clearly delineated the tumor and the thyroid. The simultaneous injection of 1 mg/kg body weight of KKD (blocking, right) decreased the metabolic trapping rate in the tumor and the thyroid. The further effects were the higher background by the elevated blood level and decreased kidney uptake.

The 10c biodistribution and -kinetics was also studied by small animal PET (Figures 9–11). The tumors (PC-3, DU-145) were clearly visible in these representative images (Control). However, the activity distribution in the tumors was relative heterogeneous following the heterogeneity of the tumor tissue. An additional reason is the high interstitial pressure in the tumor center decreasing the perfusion. The highest levels of 10c were in the liver and kidneys. In the most PET studies, also the thyroid gland was visible, which could not be measured in the extractive biodistribution experiments. This was surprising because of the very small size of the thyroid. The simultaneous injection of KKD with the 10c (lower part of Figure 9) reduced the activity concentration also in this tissue. In contrast to the biodistribution, the 10c uptake in stomach could not be studied, because of the close anatomical position of the stomach to the liver and kidneys with its high activity uptake. The stomachs relative thin wall caused a spill with the surrounding tissues that didn´t allow clear visualizing of the stomach in the PET images.

The Figure 10 shows the kinetics of 10c. At the end of the measurement, at two hours after injection we measured the activity concentration in the PC-3 tumors [2.02 ± 0.28 SUV, (Control) and 0.99 ± 0.05 SUV (blocked)] and in the DU-145 tumors [1.34 ± 0.24 SUV (Control) and 0.68 ± 0.05 SUV (blocked)]. The activity concentration was slightly higher in the PC-3 than in DU-145 tumors. The blocking with KKD resulted in approximately two times lower concentration of the radiotracer in both tumor tissues. The incomplete blocking to the half of the 10c uptake seems to be caused by the relative high blood level, non-specific binding in the tissue and the sink effect from the whole-body distribution of the Ghrelin receptors. In the liver the release of ^64^Cu^2+^ from the NODAGA-complex [51] could. Similar effect of the competition with KKD showed the thyroid time–activity curve. The very low mass/volume (6 μL) of the thyroid in mice influenced the measured activity concentration by spillover with the surrounding tissue and low recovery of the PET signal. In contrast to the tumor and thyroid the blood concentration of 10c was increased after KKD injection, which also affected the blood clearance. The half-life of the 10c in the Control was 7.9 min and in the KKD blocked animals 23.6 min. This was caused by the competition of KKD not only on the GHS-R1a but also on binding sites in the organism important for the elimination of 10c that increased the blood background in the “blocked” animals. Exemplarily parametric metabolic trapping rate images (Patlak K_m_) were calculated from the blood curves and the dynamic PET images and are presented in Figure 11. This shows how the PET study with 10c allows for the modelling of whole-body tracer kinetics by directly estimating the metabolic rate constant based on a common irreversible two-compart kinetic model. In this case, the Patlak K_m_, image allows for the demonstration of the metabolic trapping that is dependent on the GHS-R1a density of the tissue and the heterogeneity of the distribution in the tumor. The blocking effect on the thyroid K_m_ also demonstrated the presence and the GHS-R1a dependent radiotracer accumulation in this organ. A limitation of the parametric imaging was the use of the whole blood activity and not of the arterial blood. However, the slow tissue extraction of the 10c lowered this effect. The other limitation of this method in our example is that the tracer accumulation was not irreversible. It could be demonstrated that the 10c allows quantitative imaging of the Ghrelin receptor distribution within the organism.

### Immunohistochemistry of GHS-R1a in tumors and stomach of the studied mice

**Figure 12:**
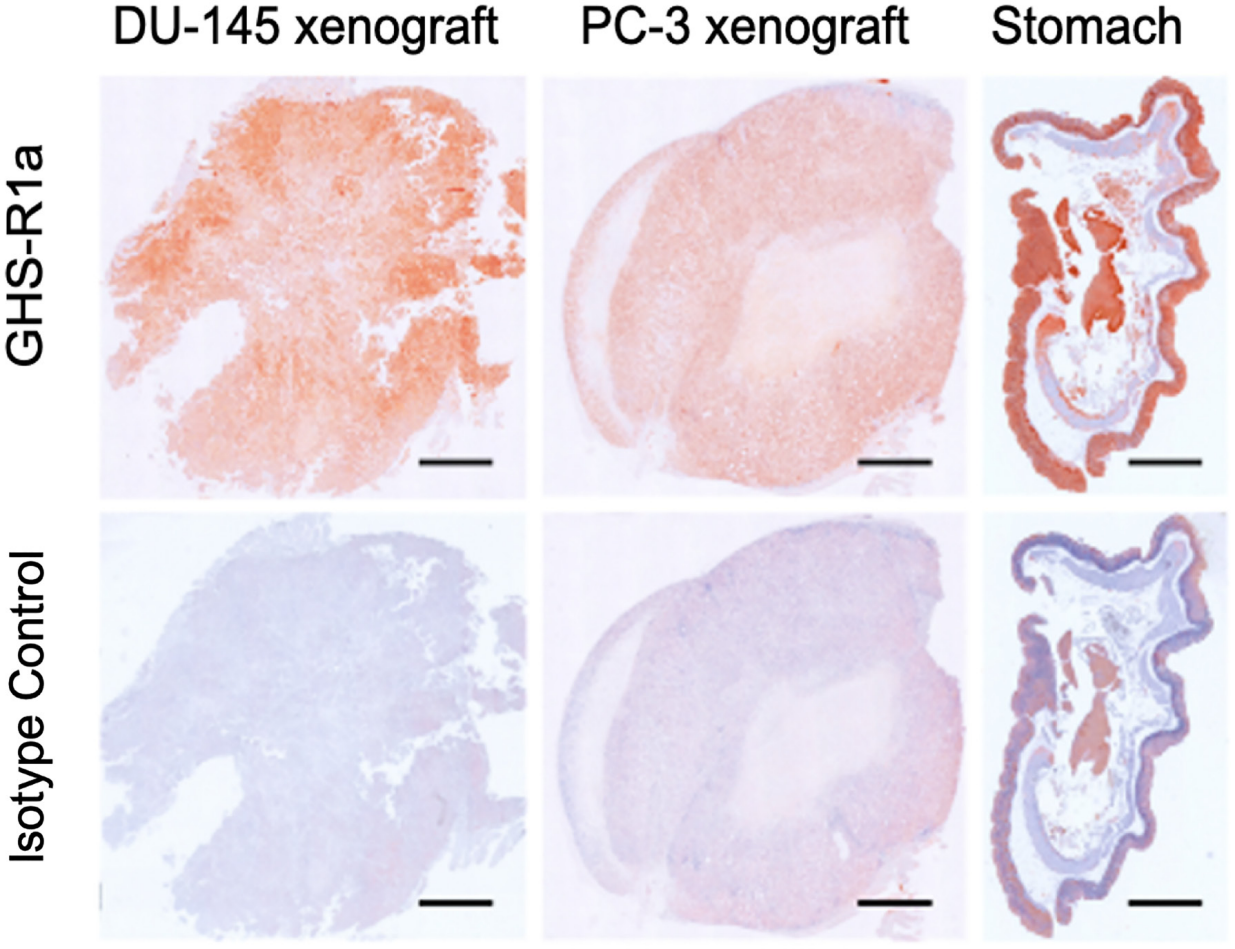
Immunohistochemistry of GHS-R1a. Immunohistochemistry of GHS-R1a in DU-145 and PC-3 xenografts mouse compared to mouse stomach; scale bar: 1 mm.

To validate the presence of GHS-R1a in the tumors and mice studied an independent technology was applied. The immunohistochemistry of human prostate cancer xenografts in NMRI-Foxn1nu/nu mice showed that GHS-R1a was expressed in both DU-145 and PC-3 tumors (Figure 12). Mouse stomach also expressed the GHS-R1a and served as positive control.

### Immunoblotting of GHS-R1a in tumors and stomach of the studied mice

**Figure 13:**
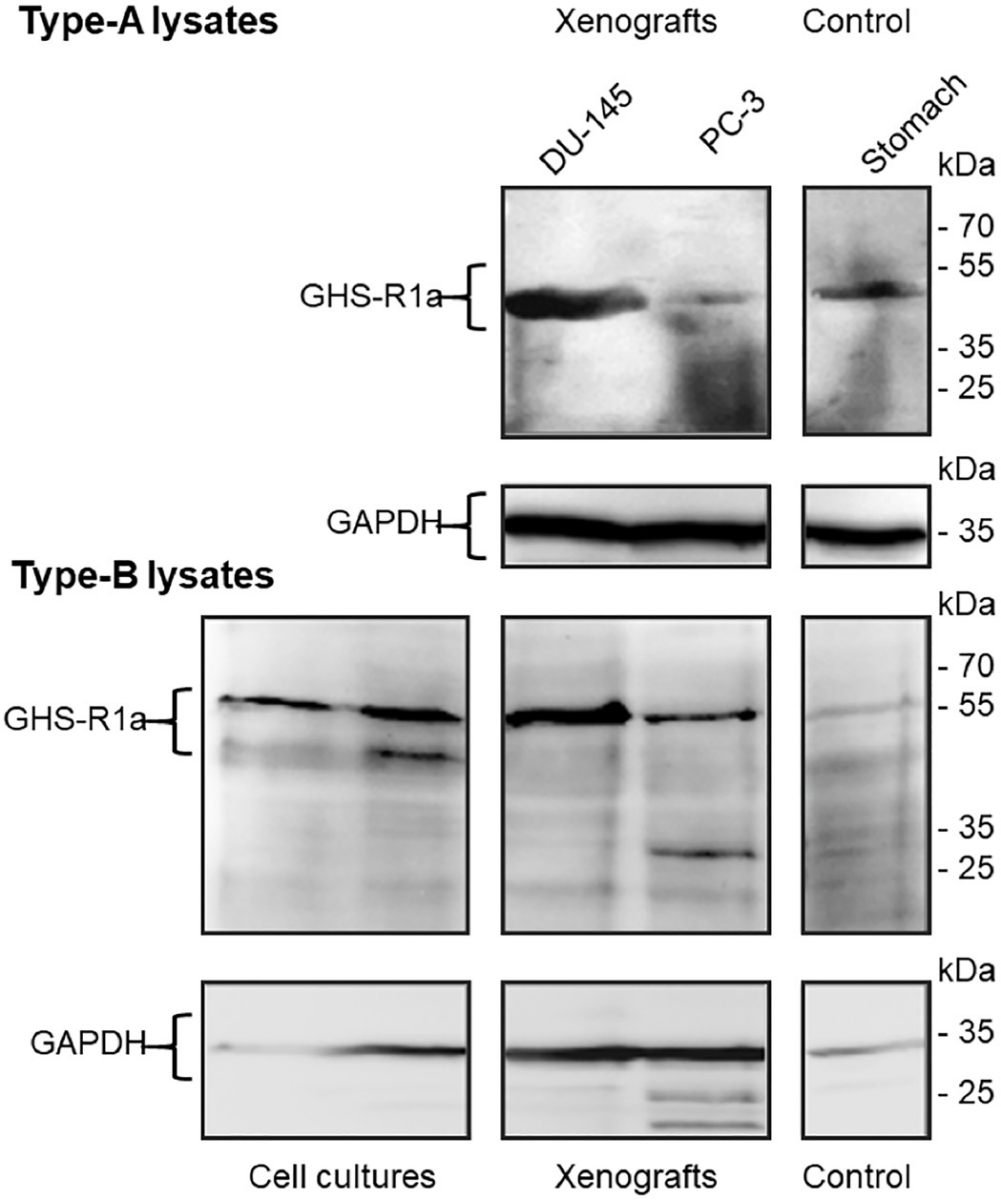
Immunoblots of GHS-R1a in lysates of DU-145 and PC-3 cell cultures, xenografts and Control. Type-A samples were heated to 100°C for 10 min and GHS-R1a was detected using the primary antibody ab170690; type-B samples were incubated at 37°C for 10 min and GHS-R1a was detected using the primary antibody ARG-031; mouse stomach served as positive control. (GAPDH) glyceraldehyde 3-phosphate dehydrogenase served as loading control.

The corresponding immunoblots confirmed the histology data showing GHS-R1a immunoreactivity in lysates of both DU-145 and PC-3 xenografts (Figure 13). Of note, GHS-R1a was not detectable using the ab170690 antibody in type-A cell culture lysates (heated to 100°C, data not shown). However, different processing of type-B lysates (no heating) and use of the AGR-031 antibody allowed for detecting GHS-R1a in both cell cultures and xenografts. Considering the intensities of glyceraldehyde 3-phosphate dehydrogenase (GAPDH) loading controls, GHS-R1a levels were comparable in DU-145 and to PC-3 lysates, irrespective of sample processing or primary antibody.

The immunochemical characterizations showed the expression of the Ghrelin receptor in the tumors and stomach and are in a good agreement with the radiotracer *in vivo* accumulation in these tissues.

## DISCUSSION

The main objectives of this work were to design potent Ghrelin receptor inverse agonist radiotracers in order to evaluate: a) their biodistribution, biokinetics, and metabolism *in vivo*; b) their imaging by small animal PET and, finally; c) their possible use for mapping the Ghrelin receptors in healthy tissues and tumors. All conjugates were designed based on the structure of the peptide tracer [^68^Ga]Ga-NODAGA KwFwLL-NH_2_ 1 and the Ghrelin receptor inverse agonist K-(D-1-Nal)-FwLL-NH_2_ 2, that were previously developed in our group [47, 49, 52]. Prior to the tracer design, a SAR study was performed on the lead inverse agonist K-(D-1-Nal)-FwLL-NH_2_ 2 in order to study advantages of palmitoylation and PEGylation for further development of therapeutic and/or imaging agents. Introduction of an extra *N*-terminal lysine to the hexapeptide sequence and its subsequent palmitoylation has been achieved without loss of potency (3: KK-(D-1-Nal)-FwLL-NH_2_, 6: Palm-KK-(D-1-Nal)-FwLL-NH_2_). In contrary, substitution of Leu^6^ into Lys^6^ resulted in a drastic drop of potency whereas subsequent palmitoylation at *N*^ε^-Lys^6^ conducted to the most potent inverse agonist K-(D-1-Nal)-FwLK(Palm)-NH_2_ 5. On the other hand a loss in potency was observed when PEGylation was performed both at *N*^ε^-Lys^6^ or *N-*ter-Lys^1^ (7: K-(D-1-Nal)-FwLK(PEG2)-NH_2_; 8: PEG2-KK-(D-1-Nal)-FwLL-NH_2_). Loss of activity was often reported for PEGylated analogues of bioactive molecules and predictive power of cell-based assays for *in vivo* therapeutic effect is even discussed [53]. Nevertheless, in the best case, the PEGylated inverse agonist exhibited a 37-fold shift in potency. Although small (2 kDa), the introduction of this PEG moiety probably changed too drastically the relative low mass of the inverse agonist (ca 1 kDa) and cleavable PEG-moieties might be an approach in future studies [54].

In order to design inverse agonist tracers, the influence of single introduction of [^nat^Ga]Ga-NODAGA was first studied. Placed at the *N*-terminus of the hexapeptide 2, the bifunctional chelator decreased the potency 10-fold, although maintained in the nanomolar range (9a: [^nat^Ga]Ga-NODAGA-K-(D-1-Nal)-FwLL-NH_2_). Nevertheless, a 10-fold gain in potency was obtained compared to the first inverse agonist chelate [^nat^Ga]Ga-NODAGA-KwFwLL-NH_2_ 1 (EC_50_ = 624.0 nM). Interestingly, addition of an extra lysine linker was beneficial (10a: [^nat^Ga]Ga-NODAGA-KK-(D-1-Nal)-FwLL-NH_2_) whereas the connection of [^nat^Ga]Ga-NODAGA at the *N*ɛ-Lys^1^ was disadvantageous and decreased the potency (11a: K[NODAGA(Ga)]-KK-(D-1-Nal)-FwLL-NH_2_). Dual functionalization with palmitoyl/PEG and [^nat^Ga]Ga-NODAGA moieties, either both at the *N-*terminal lysine linker or at the *N-* and *C-*terminal lysine influenced potency in a similar manner than previously observed for peptides 5–6. Hence, as expected, a complete loss of potency was observed for the PEGylated chelate 14a, excluding its use as potential radiotracer. In contrast, palmitoylation improved potency of tracers when performed at *N*^ɛ^-Lys^1^ (12a: [[^nat^Ga]Ga-NODAGA-K(Palm)-K-(D-1-Nal)-FwLL-NH_2_), *N*-terminus (13a: Palm-K[[^nat^Ga]Ga-NODAGA]-K-(D-1-Nal)-FwLL-NH_2_) or *N*^ɛ^-Lys^7^ (15a: [[^nat^Ga]Ga-NODAGA]-KK-(D-1-Nal)-FwLK(Palm)-NH_2_ and 16a: K[[^nat^Ga]Ga-NODAGA]-K-(D-1-Nal)-FwLK(Palm)-NH_2_). Nevertheless, the competitive binding study showed that palmitoylation was preferred at the *C*-terminal Lys^7^ (15a). Interestingly, the position of [^nat^Ga]Ga-NODAGA was more critical and only tolerated in the *N*-terminal region (15–16a versus 17–18a).

Inverse agonist chelates 9 and 10 and the palmitoylated analogue 15 were selected for *in vivo* evaluation. Radiolabeling with [^68^Ga]Ga^3+^ and [^64^Cu]Cu^2+^ was performed in mild conditions and tracers 9b-c, 10b-c and 15b-c were obtained with high radiochemical purity and high specific activity. Elevated concentrations of all tracers were observed in peripheral tissues, supported by high levels in blood and a steady decrease up to half of their initial concentration within one hour after injection.

Interestingly, radiotracers 9b-c and 10b-c structurally differ in one *N*-terminal lysine linker and exhibited a similar biodistribution in most organs. The palmitoylated tracers 15b-c showed a distinct behavior with higher concentration in blood, spleen, and brain and an almost exclusive hepatic accumulation. However, this effect is not fully understood; it can be both the higher receptor binding affinity of 15a, 15b, 15c and, or in combination, their higher lipophilicity.

Choosing the appropriate chelator and radiometal is critical for tracer optimization [55, 56]. Gallium ([^68^Ga]Ga^3+^) and copper ([^64^Cu]Cu^2+^) have indeed different characteristics that can influence *in vivo* behavior. [^68^Ga]Ga^3+^-tracers have a physical half-life of 68 min and maintain the original charge of the compounds and *in vivo* studies could be extended up to 4 hours. In contrast, [^64^Cu]Cu^2+^-tracers possess a physical half-life of 12.07 h that allows PET studies up to two days but add a negative charge to the compounds. In addition, copper complexes are larger than gallium complexes due to the larger ionic radius of the copper ion (87 versus 76 pm). At last, the higher positron energy of [^68^Ga]Ga^3+^ results in lower spatial resolution of the PET images (β^+^ = 89% versus 18%). In this study, biodistribution of [^68^Ga]Ga^3+^- and [^64^Cu]Cu^2+^-chelates were comparable 5 minutes after injection except in blood where uptake of ^68^Ga-15b was higher than ^64^Cu-15c and in the spleen where the contrary was observed (^64^Cu-15c > ^68^Ga-15b). After 60 minutes, concentrations of [^68^Ga]Ga^3+^-tracers were higher in most organs than [^64^Cu]Cu^2+^-tracers, with two exceptions: ^64^Cu-15c in the spleen and ^64^Cu-9c in intestine. This discrepancies are probably due to the lower charge of the [^68^Ga]Ga^3+^-NODAGA complex. Another important organ represents the liver where free copper is highly accumulated by binding to the superoxide dismutase [57]. Comparing the biodistribution of 10b and 10c in healthy Wistar rats with published data shows that the liver uptake of both radiotracers is relatively fast and increased only slowly, and the ^68^Ga compound had a similar or slightly higher liver uptake than the ^64^Cu labeled compound. This demonstrates that the liver uptake is primarily caused by the Ghrelin derivative itself but not released from the copper NODAGA complex.

On the other site, in the blood only traces of the radioactive metabolites were overserved, which indicates that radioactive metabolites were mostly retained in the liver and kidneys and were eliminated. This behavior allows to use the blood activity concentration in the PET directly for quantitation and does not need metabolite corrections.

It is important to mention that accumulation in the kidney was often reported for tracers functionalized with chelators and highly depend on the chelator structure [58]. In this study, however, the low renal accumulation of all chelates supports the choice of NODAGA and its use for imaging [59–62]. In contrast, all tracers exhibited a predominant hepatobiliary elimination, especially the palmitoylated analogues, which correlated with their lipophilicity and their moderate size (MW = 1326 to 1708 Da). The similar liver uptake of both ^64^Cu- and ^68^Ga-radiotracers seems to be primarily caused by the Ghrelin radiotracers itself and not by copper release from the chelate complex with NODAGA [57, 63, 64].

Accumulation in brain is a central parameter as most prominent levels of Ghrelin receptors are located in the central nervous system and Ghrelin derivatives can excite central effects at therapeutic dose [1, 8, 65–67]. Nevertheless, it is not yet clear how the receptor is activated in the brain and two hypotheses are currently discussed. Circulating Ghrelin may directly enter the CNS through permeable area of the blood brain barrier or could indirectly activate its receptor via the vagus nerve [8, 9]. However, tracer concentrations in blood are about six to nine orders of magnitudes lower than therapeutic dose. In addition, the time of interaction in the brain is limited by the effective half-life of the tracer in which the physical half-life of the radionuclide used, and the biological half-life of the tracer have to be simultaneously considered. Additionally, the brain uptake is limited by the penetration of the tracer through the blood-brain barrier. In this study, specific brain accumulation of all tracers have been detected and the total uptake of 15b, according to its molar activity, reached 19 nmol × kg^-1^ brains after one hour. This concentration is similar to EC_50_ (18 nM) and Ki (11 nM) of 15a *in vitro*. Based on this estimation and on the metabolic stability of the compound, it can be hypothesized that the level of 15b is sufficient to interact with the Ghrelin receptor system in the brain.

Both, the palmitoylated analog 15b that was radiolabeled with short lived ^68^Ga and the non-palmitoylated 10c that was radiolabeled with ^64^Cu were identified as suitable PET imaging probes for the Ghrelin receptor. ^68^Ga is the preferable radionuclide for human application of low molecular weight radiopharmaceuticals. On the other site ^64^Cu is the radiometal of choice for preclinical applications of low molecular weight radiometal tracers, because it yields the highest resolution in PET imaging and the physical half-life allows longer study times for metabolite analysis at later time points. Easy synthesis of 10c and its structural characterization, its small molecular weight, good tumor penetration, and the long physical half-life were the major reasons to select 10c for the preclinical prostate cancer imaging in mice.

The biodistribution and metabolism of the radiotracers are primarily dependent on the bioavailability and potential competitors in the organism. The total amounts of injected peptide and the resulting blood plasma concentrations in the biodistribution studies of the ^64^Cu-labeled peptides were comparable with reported Ghrelin level *in vivo* (0.09–0.22 nM) [33–35] of lined rats.

The accumulation of 10c in mostly all organs with the exception of the low accumulation in the skeletal muscles is well in concordance with the literature about the distribution of the GHS-R1a in humans and in other mammals [15, 68, 69]. The biodistribution studies using male mice with xenotransplanted prostate tumors are in good agreement with the broad functional expression of GHS-R in many organs and tissues. Exemplarily PC-3 and DU-145 tumors and as example of the normal tissue, the stomach, were evaluated by immunohistochemistry and Western Blots for expression of the GHS-R1a. The GHS-R1a protein was in all three tissues clearly visualized. The GHS-R1a dependent biodistribution of 10c was supported by the relative high uptake of this ^64^Cu-labeled Ghrelin derivative with SUVs larger than 0.3 and by the complex pattern of inhibition effects with KKD. The highest blocking effect was found in testes, brain, tumor and stomach, which are all organs with a known high expression of GHS-R [6, 7]. The broad distribution of the Ghrelin binding sites in the organism caused an effect that is known from lipophilic receptor ligands with many binding sites, the simultaneous injection of a blocking drug increased the radiotracer accumulation on target sites. The mechanism behind this is the increased availability of the radiotracer that can be delivered from many binding sites and when it is released then it can bind to sites either with higher binding affinity or higher density in the tissue. An example is the increase and longer half-life of 10c in the blood and the observed increase of the 10c in the adrenals after blocking by KKD.

The data support that the studied radiometal Ghrelin inverse agonists could be used for the imaging of functional expression of Ghrelin receptors *in vivo* that these radiotracers have the potential to gain a deeper understanding on Ghrelin and its receptors *in vivo* in health and disease, in particular, in cancer [31]. The Ghrelin binding, constitutively active growth hormone secretagogue receptor type 1a (GHS-R1a) has been imaged and quantified by the developed novel compounds, structurally based on the potent inverse agonist KK-(D-1-Nal)-FwLL-NH_2_ that were evaluated for receptor binding and efficacy *in vitro*. The radiotracers were specifically accumulated in xenotransplanted human prostate tumor models (PC-3, DU-145) in mice. The tumors were clearly delineated by PET. According to the publication of Charron et al. [44] about one hour after injection the tumor uptake of the studied ^68^Ga-compound was 0.25 SUV. In the biodistribution experiments the 10c reached at two hours a 2.68 ± 1.11 SUV in the tumor and could be blocked by Ghrelin and KKD to 1.28 ± 0.22 SUV and 1.26 ± 0.34 SUV, respectively. This shows an approximately 10 times higher specific tumor uptake of 10c. The other large difference was the high kidney uptake of the compound of Charron et al. in comparison to the 10c radiotracer that accumulated much higher in the liver. In addition, the biodistribution 10c in rats and mice showed specific accumulation of the radiotracers in GHS-R1a expressing tissues, like stomach and thyroid in which the radiotracer uptake has been partially blocked by KKD. In the arterial blood plasma only the original compounds were practically found. This allowed us to use the time activity concentration curve without metabolite analysis. The relatively slow extraction of 10c by the tissues also allowed us to use the blood AUC measured over the vena cava. The potential bias is similar for both, the control and blocked animals by KKD. For comparison the Patlak *K*_m_ images were estimated using the ROVER software and the effects of blocking visualized. The [^64^Cu]Cu- or [^68^Ga]Ga-NODAGA-NH-K-K-(D-1-NaI)-F-w-L-L-NH_2_ radiolabeled inverse agonists with respect to its relative simple synthesis, high affinity and medium efficacy, high metabolic stability, and the suitable pharmacokinetic profiles, seem to be potent, and safe leads of future imaging agents for visualization and quantitation of GHS-R1a expression in normal and cancer tissues by PET. The GHS-R1a *in vivo* imaging and quantitation has the potential to serve as biomarkers of the Ghrelin system in precision medicine.

## MATERIALS AND METHODS

### Materials

Fmoc amino acid derivatives, diisopropylcarbodiimide (DIC), 1-hydroxybenzotriazole (HOBt), piperidine, Rink amide resin (L = 0.7 mmol × g^-1^), were purchased from Iris Biotech (Marktredwitz, Germany) or Novabiochem (Läufelfingen, Switzerland). If not specified, the side chain protecting groups are *t*Bu for Glu, Ser, Thr, and Tyr; Boc for Lys and D-Trp; Trt for Gln; and His and Pbf for Arg. Thioanisole, *p*-thiocresol, ethandithiol, piperidine, hydrazine monohydrate, 4-dimethylaminopyridine (DMAP), triisopropylsilane (TIS), and *tert*-butanol was purchased from Fluka (Taufkirchen, Germany). Dichloromethane (DCM) and *N*,*N*-dimethylformamide (DMF) were purchased from Biosolve (Valkenswaard, Netherlands). Palmitic acid, Trifluoroacetic acid (TFA), acetic anhydride, diisopropylethylamine (DIPEA), octanoic acid, and Ga(NO_3_)_3_ were obtained from Sigma-Aldrich (Taufkirchen, Germany). NODAGA(tBu)_3_ (4-(4,7-bis(2-*tert*-butoxy-2-oxoethyl)-1,4,7-triazonan-1-yl)-5-*tert*-butoxy-5-oxopentanoic acid) was obtained from CheMatec (Dijon, France). Gradient-grade high-performance liquid chromatography (HPLC) solvent acetonitrile (ACN) was from VWR (Darmstadt, Germany). All reagents and solvents were used without purification as provided from the commercial suppliers. Non-modified Ghrelin used as a control in biological assays was obtained from PolyPeptide (Hillerød, Denmark). For cell culture and *in vitro* assays, Dulbecco’s phosphate buffered saline without calcium and magnesium (PBS), DMEM, high glucose (4.5 g × L^–1^) with l-glutamine, heat-inactivated fetal bovine serum (FBS), penicillin/streptomycin, and trypsin/EDTA (1:250) were purchased from PAA (Pasching, Austria). Metafectene was purchased from Biontex (Martinsried, Germany). [^3^H]-myo-inositol (681 MBq × mmol^–1^; 25.0 Ci × mmol^–1^) was from GE Healthcare Europe GmbH (Braunschweig, Germany). Cell culture flasks (75 cm^2^) and 24-well plates were from TPP (Trasadingen, Switzerland).

### Instruments

Automated peptide synthesis was performed with a multiple peptide synthesizer (Syro, MultiSynTech, Bochum, Germany). Preparative and semi-preparative HPLC were performed using a Shimadzu system on a Phenomenex RP18-column (21.4 × 250 mm; 10 μm/90 Å). Analytical HPLC were performed using a Merck-Hitachi system with a Phenomenex Jupiter 4u Proteo 90 Å (250 × 4.6 mm; 4 μm; 90 Å). All peptides were analyzed by matrix-assisted laser desorption ionization–time-of-flight (MALDI-ToF) using a Bruker Daltonics Ultraflex III mass spectrometer. The 1.85 - GBq (50 - mCi) ^68^Ge/^68^Ga-generator was purchased from iThemba Laboratories with the ^68^Ge on a SnO_2_-cartrige and eluted according to the manufacturer’s recommendations using a remote-controlled module.

### Synthesis of Ghrelin inverse agonist derivatives and conjugation with NODAGA for labeling with metal-isotopes

The synthesis of the peptides was performed on a Rink amide resin (13.5 μmol) with an automated peptide synthesizer and following a Fmoc/t-Bu strategy as previously described [52]. Special amino acids were coupled manually. NODAGA(tBu)_3_ was introduced after selective cleavage of Mtt or Fmoc protecting groups [46]. Palmitic acid and PEG2 were coupled on precursors b and d (scheme 2) after selective cleavage of Mtt or Fmoc protecting groups. The resins were swollen in 500 μL of DMF for 15 min. For palmitoylation, palmitic acid (0.15 mmol, 10 equiv) and HOBt (0.15 mmol, 10 equiv) were dissolved in 200 μL DMF and the solution was added to the resin with DIC (0.15 mmol, 10 equiv). For PEGylation, MeO-PEG2-NHS (0.045 mmol, 3 equiv) and DMAP (0.09 mmol, 6 equiv) were dissolved in 200 μL DMF and the solution was added to the resin with DIC (0.09 mmol, 6 equiv). Reaction mixtures were shaken overnight at room temperature, resins were then successively washed five times with DMF, DCM, MeOH, Et_2_O, and dried *in vacuo*. Completion of the coupling reaction was monitored with a Kaiser test.

### Complexation with natural gallium

Complexation with natural gallium of NODAGA-peptide conjugate 9–18 was performed for *in vitro* assays following established procedure and yielded NODAGA(Ga)-peptide chelates 9–18a [70]. Complexation of conjugates 9–18 with natural ^nat^Ga^3+^ was performed by incubating the peptides with a solution of ^nat^Ga(NO_3_)_3_ in acetate buffer (pH 5) at 37°C [46]. The chelates 9–18a (scheme 2) were directly purified by HPLC to remove the excess of metal and used for *in vitro* assays.

### Radiolabeling with ^68^Ga

TFA-HCl exchange and radiolabeling with ^68^Ga was also performed on chelates 9, 10 and 15 according to the procedure already described and led to 9b, 10b and 15b [46].

### Radiolabeling with ^64^Cu

The production of ^64^Cu was performed at a PET cyclotron, following standard procedures [70]. The peptide conjugates 9, 10 and 15 were radiolabeled by adding 50 – 200 MBq of [^64^Cu]CuCl_2_ pH 5.5 that was adjusted with 2 M NH_4_-acetate to approximately 20 nmol peptide dissolved in 100 μL water to afford 9c, 10c and 15c. The radiochemical purity and integrity were confirmed by Radio-HPLC. Before formulation for the *in vitro* or *in vivo* application, the reaction mixture was filtrated (45 μm pore size, REZIST 13/0.45 PTFE, Schleicher & Schuell, Dassel, Germany). The filtrates were then diluted with isotonic sodium chloride solution E-154 (154 mmol × L^–1^ Na^+^, 154 mmol × L^–1^ Cl^-^, Serumwerk Bernburg, Germany) to reach concentration of about 20 MBq × mL^-1^ and directly used for the radiopharmacological studies. The acetate concentration in the final formulation did not exceed 150 mmol × L^-1^ and was suitable for intravenous injection.

### Inositol triphosphate turnover assay

Inositol triphosphate turnover assay to measure the potency of peptide 3–8 and 9a-18a was performed in COS-7 cells stably or transiently transfected with the Ghrelin receptor, as previously described in detail [46, 49]. Data were analyzed with GraphPad Prism 6.0 program (GraphPad Software, San Diego, CA, USA). EC_50_, pEC_50_ and *E*_max_ values were obtained from concentration–response curves. All signal transduction assays were performed in duplicate and repeated at least two times independently.

### Competitive binding assay

Competitive binding assay to measure the affinity of 10a, 12a and 15a was performed in COS-7 cells stably transfected with the Ghrelin receptor, as previously described [49]. Each experiment was performed in triplicates. IC_50_ values of the binding curves were calculated by nonlinear regression on a sigmoidal dose-response based model by using program GraphPad Prism 6.0. *K*_i_ values were calculated by Cheng-Prusoff equation.

### Animals, feeding, husbandry, and animal preparation

Animal experiments in male Wistar rats (Wistar Unilever, HsdCpb:WU, Harlan Winkelmann, Borchen, Germany) (5–7 week old) and in male NMRI nu/nu mice (7–14 week old) were carried out according to the guidelines of the German Regulations for Animal Welfare. The protocol was approved by the local Ethical Committee for Animal Experiments (reference number 24-9168.21-4/2004-1).

Rats and mice were housed in separate rooms under standard conditions with free access to food and tap water.

For the generation of subcutaneous tumors PC-3 (ATCC^®^ CRL-1435™) and DU-145 (ATCC^®^ HTB-81™) cells were used. The NMRI nu/nu mice were subcutaneously xenotransplanted into the right legs with these cells according to the published protocol [71, 72].

### Measurement of original compounds and radioactive metabolites

The metabolite analysis in blood samples was carried out on male Wistar rats that were anesthetized with desflurane. The guide value for breathing frequency was 65 breaths × min^-1^. Animals were put in the supine position and placed on a heating pad to maintain body temperature. The spontaneously breathing rats were treated with 100 units × kg^–1^ heparin (Heparin-Natrium 25.000-ratiopharm, Ratiopharm, Germany) by subcutaneous injection to prevent blood clotting on intravascular catheters. After local anesthesia by injection of Lignocaine 1% (Xylocitin loc, Mibe, Jena, Germany) into the right groin, catheter was introduced into the right femoral artery (0.8 mm Umbilical Vessel Catheter, Tyco Healthcare, Tullamore, Ireland) for blood samples for metabolite analysis, for gas analysis, arterial blood pressure measurements and a second needle (35 G) catheter into one tail vein was used for administration of the 9b, 9c, 10b, 10c, 15b and 15c. Arterial blood samples were collected at 1, 3, 5, 10, 20, 30, 60, and 120 min after injections and the activity (% ID × mL^–1^) was measured to give the arterial blood activity concentration. If the activity concentration in the blood was larger than 1 kBq × mL^-1^ then the sample was used for further evaluation. Blood cells were separated by centrifugation (5°C, 5 min, 8000 rpm), and plasma proteins were precipitated using 60% acetonitrile and subsequent centrifugation (5°C, 5 min, 8000 rpm). The supernatant was analyzed by radio-HPLC. The radio-HPLC system (Agilent 1100 series) applied for metabolite analysis was equipped with UV detection (254 nm) and an external radiochemical detector (Ramona, Raytest GmbH, Straubenhardt, Germany). Analysis was performed on a Zorbax C18 300SB (250 × 9.4 mm; 4 μm) column with an eluent system C (water + 0.1% TFA) and D (acetonitrile + 0.1% TFA) in a gradient 5 min 95% C, 10 min to 95% D, and 5 min at 95% D at a flow rate of 3 mL × min^–1^. HPLC analyses were performed on 9b, 9c, 10b, 10c, 15b and 15c added to a rat blood sample (0 min), and arterial blood samples from up to 120 min after injections and on urine sample from 120 min after injection. The extracts from other tissues were prepared by Ultra-Torrax TR 150 (1000 rpm) and ultrasound homogenization as 10% solution in PBS, centrifugation (5°C, 70.000 g × min) and subsequent precipitation by 60% acetonitrile. The supernatant was analyzed by the described radio-HPLC.

### Biodistribution

Two groups of four male Wistar-Unilever rats aged between 5 and 7 weeks and weighing 186 ± 11 g (mean ± SD) for each time point were intravenously injected into a tail vein with 0.5 mL of electrolyte solutions E-154 of [^68^Ga]Ga- and [^64^Cu]Cu-peptides containing approximately 0.1 - 0.3 MBq. The molar activity was approximately 30 GBq × mmol^–1^ at the time of injection (Table 3). In blocking experiments, the radiotracers were simultaneously injected with 1 mg/kg body weight of KKD or Ghrelin. Animals were sacrificed at 5 and 60 min post injection. Blood and the major organs were collected, weighed, and counted in a Wallac WIZARD automatic gamma counter (PerkinElmer, Germany). The radioactivity of the samples was decay-corrected and calibrated by comparing the counts in tissue with the counts in aliquots of the injected radiotracer that had been measured in the gamma counter at the same time. The activity amount in organs that could be completely extracted was expressed as percent of injected dose (% ID). The activity concentrations in tissues were calculated as standardized uptake values [SUV = (activity × mL^–1^ tissue)/(injected activity/body weight), mL × g^–1^)]. The SUV was used for better comparison within animals of different size and weight and with other species and to compare the biodistribution and the PET data.

The tumor mice with xenotransplanted PC-3 or DU-145 tumors were used when the tumors reached 0.54 ± 0.34 g or 0.18 ± 0.09 g (mean ± SD, n = 9), respectively. The mice were divided in three groups of three animals and injected with 100 μL E-154 containing 0.2 MBq 10c alone (control) or simultaneously with 1 mg/kg body weight of Ghrelin or KKD for *in vivo* competition with the radiotracer. The mice were sacrificed one hour after injection and the organs and tissues were extracted. The activity measurements and calculations were carried out as described for the rat biodistribution experiments.

### Immunohistochemistry

Tissue sections were dewaxed in RotiHistol (ROTH) and re-hydrated in a graded series of ethanol (100, 96, 85, 70, 50% (v/v), H_2_O). Antigen retrieval was performed in boiling 10 mmol/L citrate buffer (pH 6.0). Endogenous peroxidase was quenched with 3% (v/v) H_2_O in Tris-bufferd saline containing 0.1% Tween 20 (TBS-T). Endogenous biotin was blocked using a commercial biotin blocking system (DAKO). Non-specific binding was blocked using 10% (w/v) fetal bovine serum in TBS-T. GHS-R1a was detected using the primary antibody ab134152 (ABCAM). Negative controls were incubated with blocking solution only. Specific binding was detected using the biotinylated secondary antibody 111-065-003 (1:200; DIANOVA) and ExtrAvidin peroxidase E2886 (1:50; SIGMA-ALDRICH). Sections were stained with 3,3′ diaminobenzidine, counterstained with eosin, and imaged using the AXIO Imager A1 microscope (CARL ZEISS).

### Immunoblotting

Cells and tissues were lyzed in ice-cold radioimmunoprecipitation assay buffer (SIGMA-ALDRICH) supplemented with 1 mM dithiotreitol, 1 mM Na_3_VO_4_, 5 mM NaF, 1 mM phenylmethylsulfonyl fluoride, and 1 μg/mL leupeptin. Tissues were disrupted using the TissueLyzer (QUIAGEN). Samples were diluted 5:1 in 5× loading buffer composed of 62.5 mol/L Tris-HCl (pH 6.0) supplemented with 10% (v/v) glycerin, 5% (v/v) β-mercaptoethanol, 2% (w/v) sodium dodecyl sulfate, and 0.01% (w/v) bromophenol blue. Samples were further processed in two different ways; type-A samples were heated to 100°C for 10 min, type-B samples were incubated at 37°C for 10 min. Proteins were separated on 10% (w/v) sodium dodecyl sulfate polyacrylamide gels and transferred to PVDF membranes (WHATMAN). Non-specific binding was blocked with Tris-HCl containing 0.5% (v/v) Tween-20 and 5% (v/v) fat-free milk. In group-A samples GHS-R1a was detected using the primary antibody ab170690 (1:200; ABCAM). In group-B samples GHS-R1a was detected using the primary antibody AGR-031 (1:500; ALOMONE LABS). Specific binding was detected using the secondary antibody A0545 (1:5000; SIGMA-ALDRICH). For loading control, glyceraldehyde 3-phosphate dehydrogenase (GAPDH) was detected using the primary antibody G8795 (1:5000; SIGMA-ALDRICH) and the secondary antibody A9044 (1:10.000; SIGMA-ALSDRICH). Immunoreactivity was visualized using SuperSignal™ West Pico/Dura substrate (LIFE TECHNOLOGIES).

### Small animal PET

Anesthetized, spontaneously breathing animals were allowed to stabilize for 10 min after preparation. The animals were positioned on a heated bed to maintain the body temperature at 37°C. The PET studies were carried out with a NanoScanPET/CT (Mediso, Hungary) or a microPET P4^®^ (Siemens preclinical solutions, Knoxville, TN, USA). The activity of the injection solution was measured in a well counter (Isomed 2000, Dresden, Germany) cross-calibrated to the PET scanners [73, 74]. A 10 min transmission scan was recorded during this time for each subject by using a rotating point source of ^57^Co (microPET). The transmission scans were used to correct the emission scan for γ-ray attenuation caused by body tissues and supporting structures; it was also used to demarcate the body field for image registration. The PET acquisition of 60 or 120 min emission scan was started and the infusion of the ^68^Ga-/^64^Cu-labeled compound was initiated with a delay of 10 s. 0.5 mL (rats) or 0.1 mL (mice) of solutions of [^68^Ga]- or [^64^Cu]-peptides were infused over 1 min (with a Harvard apparatus 44 syringe pump) into a lateral tail vein. In blocking experiments the radiotracers were simultaneously injected with 1 mg/kg body weight of Ghrelin or KKD. At the end of the experiment, the animals were deeply anesthetized and sacrificed by an intravenous injection of potassium chloride.

### Data acquisition

Acquisition was performed in 3D list mode. Emission data were collected continuously [75, 76]. The list mode data were sorted into sinograms with 32 or 38 frames (15 × 10 s, 5 × 30 s, 5 × 60 s, 4 × 300 s, 3 × 600 s, or 9 × 600 s). The data were decay-, scatter-, and attenuation-corrected. The frames from the microPET were reconstructed by Ordered Subset Expectation Maximization applied to 3D sinograms (OSEM3D) with 14 subsets, 15 OSEM3D iterations, 25 maximum a posteriori (MAP) iterations, and 1.8 mm resolution using the FastMAP algorithm (Siemens Preclinical Solutions, Knoxville, TN, USA). The voxel size was 0.07 by 0.07 by 0.12 cm. The PET images measured with the nanoScanPET/CT were reconstructed using a three-dimensional Ordered Subsets Expectation Maximization (3D-OSEM) algorithm (Tera-Tomo, Mediso Ltd., Hungary) into dynamic frames as described and with a voxel size of 0.05 cm. No correction for partial volume effects was applied. The image volume data were converted to Siemens ECAT7 format for further processing and were then analyzed using the ROVER software (ABX GmbH, Radeberg, Germany). Masks for defining three-dimensional regions of interest (ROI) were set and the ROI’s were defined by thresholding and ROI time activity curves (TAC) were derived for the subsequent data analysis. The time activity curves over the vena cava were derived from ROI determined in the first two minutes after 1 min long infusion of the radiotracers. The ROIs were so determined that no surrounding tissue was included [77]. The ROI data and TAC were further analyzed using R (R is available as Free Software under the terms of the Free Software Foundation’s GNU General Public License in source code form) and especially developed program packages (Jörg van den Hoff, Frank Hofheinz, Helmholtz-Zentrum Dresden-Rossendorf, Dresden, Germany).

### Statistical analysis

Values are expressed as mean ± SEM. The data were statistically evaluated using ANOVA or an unpaired Student’s *t*-test with Welch’s correction and an F-test to compare the variances (GraphPad Prism 6.0). Kruskal–Wallis test was performed to identify the differences between groups. All statistical testing was performed using Prism 7.0 software. Significant difference was set at ^*^ *p* < 0.05; ^**^ *p* < 0.01; ^***^ *p* < 0.001.

## Abbreviations

AgRP: agouti-related peptide
AUC: Area under the curve
Boc: tert-butyloxycarbonyl
DIC: N’,N’-diisopropylcarbodiimide
D-1-NaI: D-1-naphtylamine-2-amino-3-naphthalen-1-ylpropanoic acid
DMF: dimethylformamide
FCS: fetal calf serum
Fmoc: 9-fluorenylmethyloxycarbonyl
GHS-R: growth hormone secretagogue receptor
HOBt: 1-hydroxybenzotriazole
Mtt: 4-methyltrityl
KKD: KK-(D-1-Nal)-FwLL-NH_2_
NODAGA: 1,4,7-triazacyclononane,1-glutaric acid-4,7-acetic acid
NODAGA(tBu)3: 4-(4,7-bis(2-(tert-butoxy)-2-oxoethyl)-1,4,7-triazacyclononan-1-yl)-5-(tert-butoxy)-5-oxopentanoic acid
PEG2: diethylene glycol
SAR: structure−activity relationship
tBu: tert-butyl
PET: Positron-Emission-Tomography
NPY: neuropeptide Y
Palm: palmitoyl
RCY: radiochemical yield
TAC: Time-activity curve
TFA: trifluoroacetic acid
Trt: trityl
BAT: brown adipose tissue
WAT: white adipose tissue
w.c: with content
